# Early development of structural networks and the impact of prematurity on brain connectivity

**DOI:** 10.1016/j.neuroimage.2017.01.065

**Published:** 2017-04-01

**Authors:** Dafnis Batalle, Emer J. Hughes, Hui Zhang, J.-Donald Tournier, Nora Tusor, Paul Aljabar, Luqman Wali, Daniel C. Alexander, Joseph V. Hajnal, Chiara Nosarti, A. David Edwards, Serena J. Counsell

**Affiliations:** a Centre for the Developing Brain, Division of Imaging Sciences & Biomedical Engineering, King's College London, SE1 7EH London, United Kingdom; b Department of Computer Science & Centre for Medical Image Computing, University College London, United Kingdom

**Keywords:** ACT, anatomically-constrained tractography, CSD, constrained spherical deconvolution, CSF, cerebrospinal fluid, FA, fractional anisotropy, DGM, deep grey matter, dMRI, diffusion MRI, DTI, diffusion tensor imaging, FDR, false discovery rate, FOD, fibre orientation distribution, FS, fraction of streamlines, GA, gestational age, GM, grey matter, MRI, Magnetic resonance imaging, NDI, neurite density index, NODDI, neurite orientation dispersion and density imaging, ODI, orientation dispersion index, PMA, post menstrual age, rFA, relative fractional anisotropy, rFS, relative fraction of streamlines, rNDI, relative neurite density index, rODI, relative orientation density index, ROI, region of interest, SGA, small for gestational age, SIFT, spherical-deconvolution informed filtering of tractograms, WM, white matter, Brain mapping, Diffusion MRI, Graph theory, Newborn, NODDI

## Abstract

Preterm infants are at high risk of neurodevelopmental impairment, which may be due to altered development of brain connectivity. We aimed to (i) assess structural brain development from 25 to 45 weeks gestational age (GA) using graph theoretical approaches and (ii) test the hypothesis that preterm birth results in altered white matter network topology. Sixty-five infants underwent MRI between 25^+3^ and 45^+6^ weeks GA. Structural networks were constructed using constrained spherical deconvolution tractography and were weighted by measures of white matter microstructure (fractional anisotropy, neurite density and orientation dispersion index). We observed regional differences in brain maturation, with connections to and from deep grey matter showing most rapid developmental changes during this period. Intra-frontal, frontal to cingulate, frontal to caudate and inter-hemispheric connections matured more slowly. We demonstrated a core of key connections that was not affected by GA at birth. However, local connectivity involving thalamus, cerebellum, superior frontal lobe, cingulate gyrus and short range cortico-cortical connections was related to the degree of prematurity and contributed to altered global topology of the structural brain network. The relative preservation of core connections at the expense of local connections may support more effective use of impaired white matter reserve following preterm birth.

## Highlights

• First characterisation of preterm brain networks weighted by microstructural features.
• Preterm brain is resistant to disruptions in development of core connections.
• Peripheral connections associated with cognition and behaviour are more vulnerable.

## Graphical abstract

**Figure:**
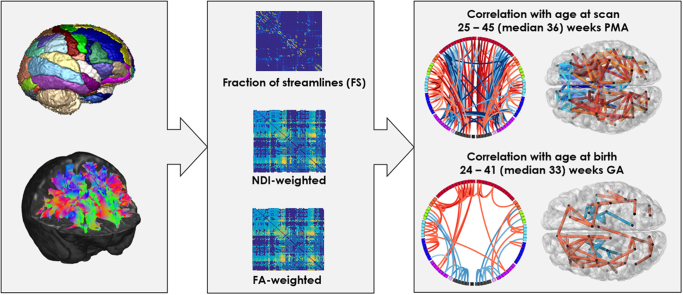

## Introduction

The third trimester of pregnancy is associated with rapid brain development including differentiation and maturation of pre-oligodendrocytes, formation of synapses between thalamo-cortical afferents and subplate neurons, axonal growth and cortical gyrification. Infants who are born preterm have a high prevalence of motor, cognitive and behavioural deficits which are evident in childhood (Bhutta et al., 2002, Saigal and Doyle, 2008) and an increased risk of developing psychiatric disorders in adulthood (Nosarti et al., 2012). The wide spectrum of disability associated with preterm birth is consistent with pervasive abnormalities in brain growth and connectivity (Back, 2015, Volpe, 2009).

By studying both brain growth and connectivity, magnetic resonance imaging (MRI) has been used extensively to improve our understanding of the neural substrate underlying neurodevelopmental impairments in this population. MR imaging studies of preterm infants have identified: reduced cortical and subcortical grey matter (Padilla et al., 2015), diminished cerebellar volumes (Limperopoulos et al., 2010) and alterations in thalamo-cortical development at term-equivalent age (Ball et al., 2013, Ball et al., 2012); changes in structural brain network topology in young children (Pandit et al., 2014) and at school-age (Fischi-Gomez et al., 2014, Kim et al., 2014); and alterations in white matter (WM) and grey matter (GM) volumes in adolescence (Nosarti et al., 2008).

Diffusion MRI (dMRI) has demonstrated altered white matter development in preterm infants without focal lesions (Anjari et al., 2007, Huppi et al., 1998), which is related to neurodevelopmental performance in early childhood (Ball et al., 2015, Counsell et al., 2008, Thompson et al., 2014) and adolescence (Groeschel et al., 2014, Northam et al., 2012a, Northam et al., 2012b). Recent studies have used dMRI to assess macrostructural connectivity *in vivo* during early development (Brown et al., 2014, van den Heuvel et al., 2015) and, using dMRI, the rich-club organisation of structural brain networks during the preterm period has been characterised, demonstrating a relative preservation of core connections at term equivalent age (Ball et al., 2014). Similar findings have been reported in preterm-born children at school-age (Fischi-Gomez et al., 2016) and in adulthood (Karolis et al., 2016). However, the impact of prematurity on weighted brain network topology prior to term equivalent age is not known, largely because it is technically challenging to obtain high quality dMRI data in the neonatal period.

Typically, connectivity strength has been derived from basic measures of the amount (percentage, or density) of streamlines connecting two regions, or alternatively, by measures of directivity such as fractional anisotropy (FA). Powerful new dMRI methods are now available, which are able to provide fibre counts that are consistent with the apparent fibre density (Pestilli et al., 2014, Smith et al., 2013, Smith et al., 2015), and a realistic estimation of neurite architecture *in vivo* (Jespersen et al., 2007, Jespersen et al., 2012, Zhang et al., 2012). For example, neurite orientation dispersion and density imaging (NODDI) (Zhang et al., 2012) is a multi-compartment model which provides quantitative measures that correlate to tissue microstructure with greater specificity than established imaging techniques, such as diffusion tensor imaging (DTI). NODDI has been used previously to characterise WM (Kunz et al., 2014) and GM (Eaton-Rosen et al., 2015) tissue characteristics during early development, and is a promising biologically interpretable (Colgan et al., 2016) alternative to anisotropy measures to weight brain connectivity (Lemkaddem et al., 2014).

In this study we used high angular resolution multi-shell dMRI to assess connectivity between brain regions using connectivity metrics obtained from the diffusion tensor (fractional anisotropy, FA) as well as streamline measures obtained using constrained spherical deconvolution (CSD) with the spherical-deconvolution informed filtering of tractograms (SIFT) algorithm (Smith et al., 2013, Smith et al., 2015) and NODDI model characteristics: neurite density index (NDI) and orientation dispersion index (ODI). We applied these technical advances for the first time in a neonatal dataset in order to assess brain development from 25 to 45 weeks gestational age (GA) using graph theory measures of structural brain networks weighted with microstructural features (NDI and ODI) and to assess the impact of prematurity on network organisation. We complemented typical network features with the assessment of core and local average connectivity characteristics, and connection-wise correlations with normal development and degree of prematurity (GA at birth), allowing us to investigate in detail the topological changes we observed.

## Methods

**Fig. 1:**
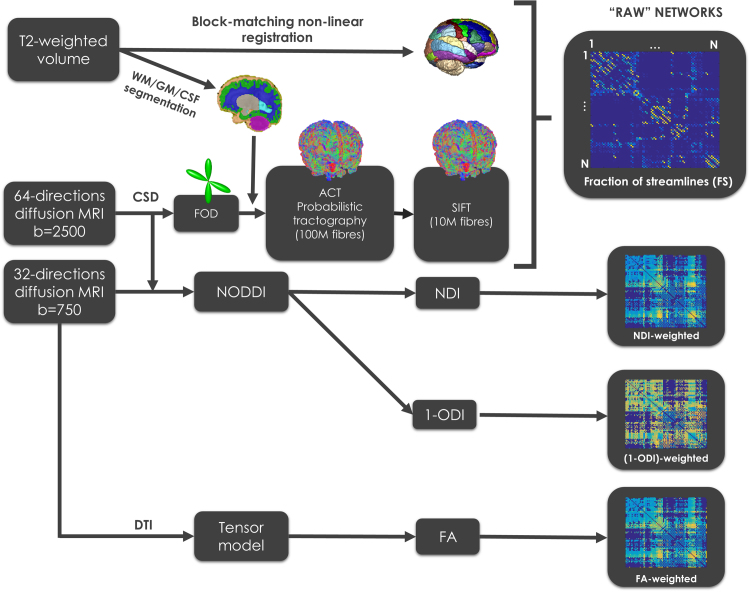
Outline of the analysis pipeline.

**Fig. 2:**
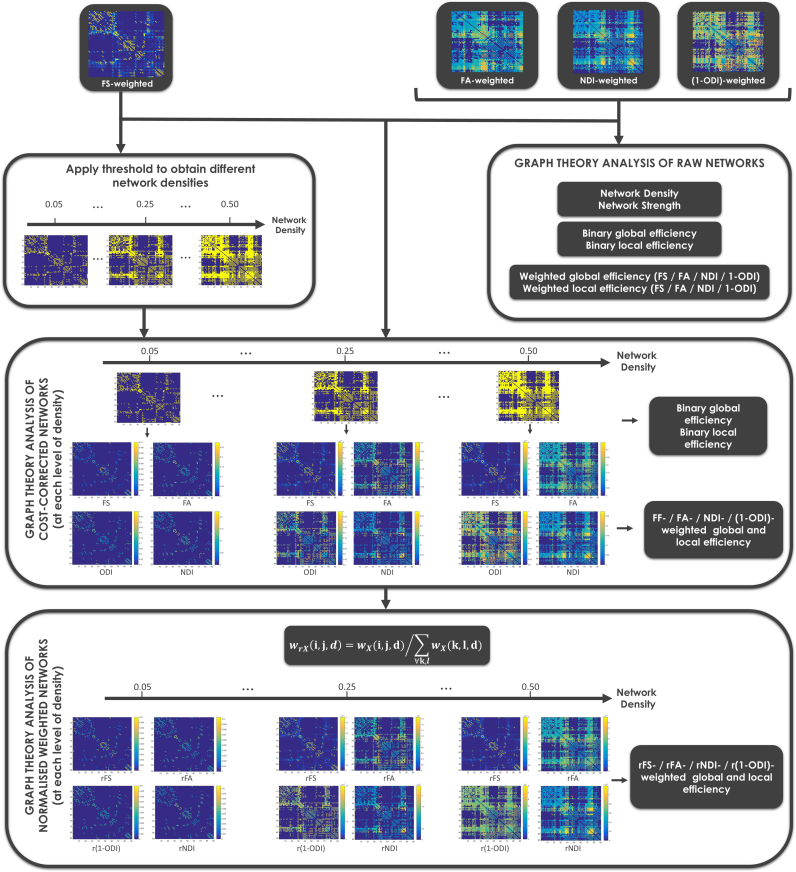
Outline of the different network normalisation approaches.

In order to investigate brain development prior to the time of normal birth and to assess the impact of prematurity on brain network organisation we performed CSD based tractography (*Tractography*), extracted network measures (*Network extraction, Network measures and Network normalisation*), determined the development of core versus non-core connections (*The development of core versus non-core connections*) and examined edge-wise correlations (*Edge-wise association in the minimum grid of connectivity*). We assessed the association between these graph theory features and edge-wise connections with age at MRI and GA at birth (*Statistical analysis*). See Fig. 1 for a scheme of the methodology used to extract brain networks and Fig. 2 for the different normalisation approaches used.

### Participants and MRI acquisition

Research Ethics Committee approval for MR imaging was granted (12/LO/1247) and written parental consent was obtained prior to MRI. The inclusion criteria for this study were MR imaging without motion artefacts, performed ≤46 weeks post-menstrual age (PMA). We studied an initial sample of 80 datasets, corresponding to 72 subjects scanned one or two times. Based on exclusion criteria of congenital malformations or evidence of focal lesions on MRI, 7 subjects were excluded, leading to a final cohort of 65 neonates with a median (range) GA at birth of 33^+2^, (range 24^+2^–41^+1^) weeks, and median PMA at scan of 36^+2^, (25^+3^–45^+6^) weeks. Of those, 8 subjects were scanned twice (median 6^+6^, range 3^+3^−8^+5^ weeks after the first scan). See Table 1 for details of perinatal clinical characteristics of the infants.

MR imaging was performed on a 3 T Philips Achieva system (Best, The Netherlands) sited on the neonatal intensive care unit using a 32-channel head coil. 3D MPRAGE (repetition time (TR)=17 ms, echo time (TE)=4.6 ms, flip angle 13°, voxel size: 0.82×0.82×0.8 mm) and T2 weighted fast spin echo (TR=8670 ms, TE=160 ms, flip angle 90°, slice thickness 2 mm with 1 mm overlapping slices, in-plane resolution 1.14×1.14 mm) were acquired. dMRI data were acquired at 2 mm isotropic resolution and SENSE factor of 2 in 2 shells; 64 non-collinear directions with a *b*-value of 2500 s/mm², 4 non-diffusion weighted images (*b=*0) with TR 9000 ms and TE 62 ms; and 32 non-collinear directions with a *b*-value of 750 s/mm², 1 non-diffusion weighted image (*b*=0) with TR 9000 ms and TE 49 ms.

A paediatrician experienced in MRI procedures supervised all examinations, and pulse oximetry, temperature and electrocardiography data were monitored. Earplugs moulded from a silicone-based putty (President Putty, Coltene, Whaldent, Mahwah, NJ, USA) placed in the external auditory meatus and neonatal earmuffs (MiniMuffs, Natus Medical Inc., San Carlos, CA, USA) were used for auditory protection. Term controls and preterm infants <37 weeks PMA at scanning were imaged during natural sleep without sedation. However, preterm infants at term equivalent age were sedated with oral chloral hydrate (25–50 mg/kg) prior to scanning.

### Pre-processing

T2-weighted brain volumes were bias corrected (Tustison et al., 2010), skull striped and tissue segmented into WM, GM, deep grey matter (DGM), cerebrospinal fluid (CSF) and cerebellum using a neonatal-specific segmentation algorithm (Makropoulos et al., 2014). Parcellation into cortical and subcortical regions was performed with a block matching non-linear registration (Tristan-Vega and Arribas, 2007) of a version of the standard anatomical automatic labelling (AAL) atlas (Tzourio-Mazoyer et al., 2002) which has been specifically adapted to the neonatal brain (Shi et al., 2011). Parcellation of the atlas was propagated into each subject's native space following the non-linear registration previously calculated and a nearest neighbour propagation. All AAL cerebellar regions were merged into one, producing a total of 91 regions in each subjects’ native space, which constituted the nodes used in the network analyses (Supplementary Table 1).

dMRI volumes were first visually inspected in order to detect data with motion artefacts, and exclude them from further analysis. All subjects included in the study had at most 8 (median 4, range 0−8) gradient directions excluded from the higher shell, and at most 6 (median 2, range 0−6) gradient directions excluded from the lower shell. Volumes were first corrected for EPI phase encoding distortions, eddy-induced distortions and subject movements by means of FSL5.0 topup-eddy algorithm (Andersson et al., 2003, Andersson and Sotiropoulos, 2015), using T2 volume rigidly registered to b0 maps and assuming a bandwidth of zero (no phase-encoding). This process was performed separately for the two acquired shells and their corresponding *b*=0 volumes, and then the lower shell was rigidly registered to the averaged *b=*0 volumes acquired with the higher shell. Gradient directions were rotated accordingly. All rigid registrations were performed with IRTK software (Studholme et al., 1999).

### Estimation of microstructural features

**Fig. 3:**
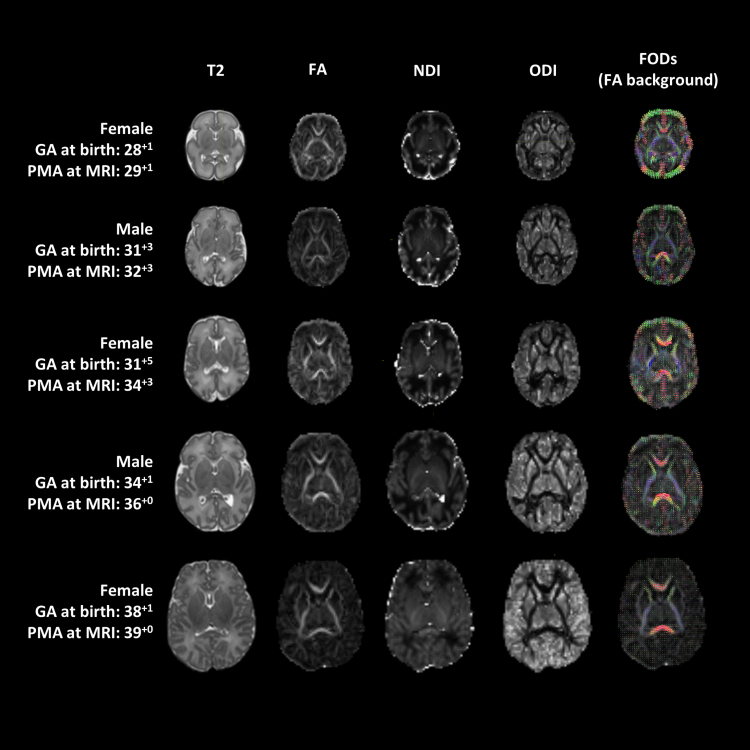
Images of five representative subjects from the study group.

Diffusion tensor imaging was fitted with *MRTrix3* (Tournier et al., 2012) on the lower shell (b=750 s/mm²) and voxel-wise FA was obtained. The NODDI toolbox (Zhang et al., 2012) provided maps of estimated intracellular volume fraction (neurite density index, NDI) and orientation dispersion index (ODI) for each subject. Data were normalised by *b*=0 volumes in order to take into account the differing TE/TR times between lower and higher shell. NODDI initialisation parameters were modified in order to better fit neonatal data by lowering the range of values considered as the fraction of the intracellular space. In addition, an in-house algorithm was used to repeat the fitting of voxels where the fitting did not converge, using AMICO, a linearized version of NODDI (Daducci et al., 2015), to provide initialization parameters. See Fig. 3 for representative figures of the infants.

### Tractography

In order to reconstruct WM fibre orientations, the fibre orientation distribution (FOD) at each voxel was obtained by means of constrained spherical deconvolution (CSD) (Tournier et al., 2007) calculated using the higher shell data only. An anatomically constrained tractography (ACT) probabilistic algorithm (Smith et al., 2012) was used to perform tractography on the FODs, with seeds on the WM/GM interface and a backtrack re-tracking algorithm, producing 100 million streamlines per subject. Those tracts where filtered using the SIFT algorithm (Smith et al., 2013), producing for each subject 10 million streamlines that best match the apparent fibre densities in the reconstructed FODs.

### Network extraction

Inference of brain networks was performed by means of an in-house algorithm combining the anatomical AAL ROIs previously parcellated with the 10 million streamlines of CSD-ACT-SIFT tractography. Each AAL ROI was considered a node of the network, considering in first instance each pair of regions *i* and *j* to be connected by a weighted edge $e_{\mathrm{ij}}$, equivalent to the number of filtered streamlines connecting both regions, not considering self-connections (i.e., $e_{\mathrm{ij}}=0,$ ∀i=j). Then, the fraction of streamlines (FS) connecting each pair of regions was defined as $w_{\mathrm{FS}}(i,j)=e_{\mathrm{ij}}/\sum_{\forall k,l}e_{\mathrm{kl}}$. Note that due to SIFT filtering, the number of streamlines will be proportional to the amount of white matter corresponding to that connection (Smith et al., 2013), and hence provides an appropriate measure of ‘connectivity’. In addition, median microstructural characteristics along streamlines connecting each pair of regions were also assessed, obtaining $w_{\mathrm{FA}}\left(i,j\right)$ (median FA), $w_{\mathrm{NDI}}\left(i,j\right)$ (median NDI) and $w_{1-\mathrm{ODI}}\left(i,j\right)$ (median 1-ODI). Note that instead of ODI, we considered 1-ODI, a measure of coherence rather than dispersion, which is a more appropriate measure to weight WM directivity,

### Network measures

Brain network characteristics were assessed by means of network density (i.e., the observed connections as a percentage of all possible connections) and total network strength (i.e., sum of all connection weights). Brain network integration capacity (the ease with which different brain regions communicate) was assessed by means of global efficiency ($E_{\mathrm{glob}}$), which is inversely related to the characteristic path length $L_{p}$. The global level of network segregation (presence of clusters, i.e., capacity for specialised processing) was assessed by means of local efficiency ($E_{\mathrm{loc}}$), defined as the average nodal efficiency, which is directly proportional to the average clustering coefficient $C_{p}$ of the network. A high level of local efficiency is also indicative of a high fault tolerance of the network to the elimination of random nodes (Achard and Bullmore, 2007).

We defined core connectivity based on the theoretical measure of edge betweenness centrality, which measures the frequency with which a connection is used in the set of shortest paths that connects all pairs of nodes. It represents a straightforward way to infer the importance of each connection in network topology (Rubinov and Sporns, 2009).

The formulations used for the graph theory features were based on the definitions compiled by Rubinov and Sporns (2009).

### Network normalisation

In order to assess the association between age at MRI and the degree of prematurity (GA at birth) on one hand and different aspects of brain connectivity and organisation on the other, we used different connectivity weights (FS, FA, NDI and 1-ODI raw networks). In order to compare the organisation of network topology among subjects, in addition to assessing the original reconstructed networks (“raw” networks), we normalised the networks obtained using three different approaches; cost-corrected networks, normalised weighted networks and comparison with random equivalents. By normalising the networks we removed the influence of the main infrastructure features (network density and total network strength) on their integration and segregation characteristics (global and local efficiency). This was done by comparing networks with the same characteristics in terms of density (cost-corrected) and total strength (normalised weighted networks), or by comparing to random equivalent networks with a comparable degree and strength distribution (random equivalents).

See Fig. 2 for a summary of the analyses performed and the different normalisation approaches.

#### Raw networks

We assessed the graph theory features of the original reconstructed networks (“raw” networks) formed by the set of connections between two regions that were linked by at least 5 streamlines, and weighted them by FS, FA and NDI and 1-ODI. This approach is comparable to that employed in previous studies (Brown et al., 2014, van den Heuvel et al., 2015).

#### Cost-corrected networks

In order to compensate for differences in network density (sparsity) between subjects, a cost-correction approach was followed (Achard and Bullmore, 2007, Wang et al., 2009), by thresholding raw networks (based on their number of streamlines) in order to obtain a desired level of network density (cost-characteristic): $w_{X}\left(i,j,d\right)=w_{X}\left(i,j\right)$ if the link between node *i* and *j* belongs to the subset of strongest connections (in terms of number of streamlines) that ensure a network density *d*, and $w_{X}\left(i,j,d\right)=0$ otherwise, where X refers to the specific weight used, i.e., FS, FA, NDI and 1-ODI. This way we obtained surrogate networks with the same level of network density in each subject. Note that the cost range was limited by the minimum value of network density for any subject included into the analysis (0.55). As there is no gold standard network density value, we calculated a set of networks for each subject corresponding to those of network density 0.05 up to 0.5 (in 0.01 steps). Graph theory features among subjects were compared at each level of network density, allowing subjects to be compared independently of their differences in network density. In order to summarise the results obtained for the whole range of network densities for each subject, we used a “cost-integrated” measure of each graph theory feature by averaging its value over the entire cost range assessed (Ginestet et al., 2011).

#### Normalised weighted networks

Even after controlling for density, the networks obtained may have different levels of total network strength (i.e., average weighted degree) between subjects. In order to compare subjects’ *weighted network topology* independently of their total network strength, we normalised the remaining weights $w_{X}\left(i,j,d\right)$ connecting each ROI *i* and *j* at each level of network density *d*, i.e., $w_{\mathrm{rX}}(i,j,d)=w_{X}\left(i,j,d\right)/\sum_{\forall k,l}w_{X}\left(k,l,d\right)$, where X refers to the specific weight used, i.e., FS, FA, NDI or 1-ODI. The normalised weight $w_{\mathrm{rFS}}$ obtained with this approach represents the relative fraction of streamlines (rFS) connecting each pair of regions at each level of network density, i.e. the percentage of streamlines connecting each pair of regions when considering only the streamlines at a given density. Analogously, $w_{\mathrm{FA}}$, $w_{\mathrm{NDI}}$ and $w_{1-\mathrm{ODI}}$ were also normalised in a similar way, representing relative FA (rFA), relative NDI (rNDI) and relative 1-ODI (r(1-ODI)). In this way, we fix both network density and average strength for all subjects, and therefore weighted graph theory features calculated from rFS-, rFA-, rNDI- and r(1-ODI)-weighted networks are independent of the subject's total network strength (and average weighted degree) at each level of network density, and so they describe the pure organisational characteristics of the networks, i.e. their *weighted network topology* (Batalle et al., 2016). We summarised the graph-theory features of normalised weighted networks by cost-integrating them over the entire cost range.

#### Alternative normalisation: comparison with random equivalent networks

Complementary to these normalisation approaches, we also computed normalised characteristic path length (α), normalised clustering coefficient (γ) and small-worldness coefficient (σ=α/γ), by comparing with matched random equivalent networks that preserve the same number of nodes and degree distributions as the real networks (Maslov and Sneppen, 2002) (Supplementary Material).

### The development of core versus non-core connections

We defined core-connections at each level of network density (from 0.05 to 0.5 at 0.01 steps) as those accounting for 50% of the total betweenness centrality in a given subject, while the rest of connections where defined as non-core (local) connections. This enabled us to test the hypothesis that the changes observed in graph theory features of normalised FA- and NDI-weighted networks are linked to a disruption in the balance of weights between core and local connections.

### Edge-wise association in the minimum grid of connectivity

In order to further characterise changes in connectivity weights associated with maturation and prematurity, individual partial correlations between age at MRI / GA at birth and FA- and NDI-weighted individual connections were assessed. To do so, for each subject we created a mask of connections accounting for a network density of 0.3 (i.e., the 30% of connections in each subject with the highest number of streamlines). Then, only connections common to all subjects were considered, creating a “minimum grid” mask for all subjects (Fischi-Gomez et al., 2014). We assessed correlations between age at MRI and GA at birth and individual edges (weighted by FA and NDI) only in this mask representing the backbone of connections present in the whole population. Visualisation of core/non-core connections and connections correlated with age at MRI / GA at birth was performed with BrainNet Viewer (Xia et al., 2013) and Circos (Krzywinski et al., 2009).

### Statistical analysis

Partial Spearman's correlations were used to assess the association between graph theory features and edge-wise connections with age at MRI and GA at birth. All statistical tests were controlled for relevant covariates using linear mixed-effects models (LME) with gender, small for gestational age (SGA), perinatal complications (necrotising enterocolitis and respiratory support) and GA at birth (or age at MRI when assessing the effect of prematurity) as fixed effects, and with a subject dependent random effect for the intercept to account for repeated measures. Edge-wise statistics were corrected using a false discovery rate (FDR) approach (Benjamini and Hochberg, 1995) controlling alpha error to 5%.

## Results

### Association between network features and age at MRI

In this section we show that increasing age at MRI was associated with changes in network density; changes in median connectivity across the network; and changes in both global and local efficiency. Assessment of pure organisational characteristics (normalised weighted networks) showed a positive association between age at MRI and rFS-weighted global efficiency, but a negative correlation with rFS-weighted local efficiency.

#### Raw network features

In the original reconstructed networks (“raw networks”), network density (ratio of actual connections over total possible) was negatively correlated with age at MRI (ρ=−0.677, p<0.001), with a range between 0.55 and 0.88 (data not shown). Median connectivity across the network, i.e., the median weight of all connections, was positively correlated with age at MRI for both FA (ρ=0.656, p<0.001) and NDI (ρ=0.684, p<0.001) and negatively correlated with 1-ODI (ρ=−0.853, p<0.001). A positive association with age at MRI was also observed in global efficiency weighted by FS (ρ=0.656, p<0.001), FA (ρ=0.547, p<0.001), NDI (ρ=0.689, p<0.001) and a negative association with age at MRI was observed when weighted by 1-ODI (ρ=−0.634, p<0.001). Similarly, local efficiency (proportional to average clustering coefficient) was positively associated with age at MRI when weighted by FS (ρ=0.610, p<0.001), FA (ρ=0.649, p<0.001) and NDI (ρ=0.688, p<0.001) and negatively associated with age at MRI when weighted by 1-ODI (ρ=0.688, p=0.002). However, it is possible that differences in network density and connectivity strengths could be driving the global and local efficiency findings.

#### Cost-corrected networks

**Fig. 4:**
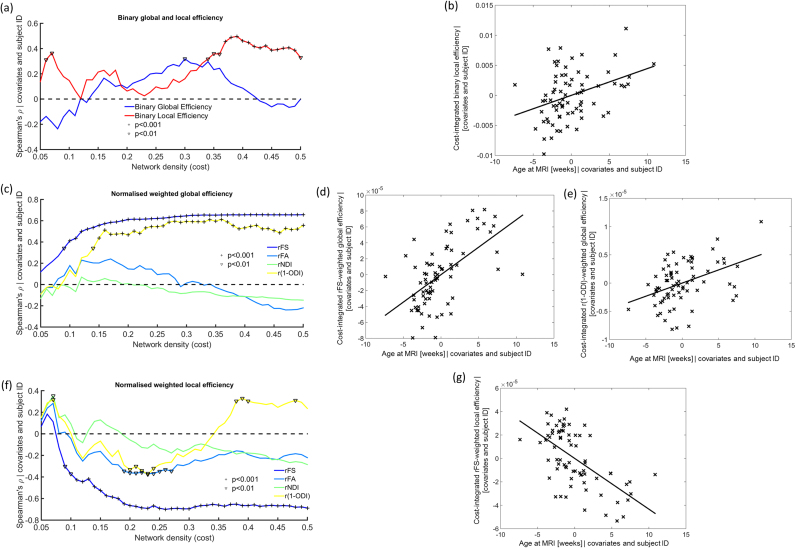
Association of graph theory features with age at MRI. (a) Spearman's partial correlation coefficient of cost-corrected binary global and local efficiency and age at MRI. (b) Residual of cost-integrated binary local efficiency vs age at MRI. (c) Spearman's partial correlation coefficient of cost-corrected weighted normalised global efficiency and age at MRI. (d) Residual of cost-integrated FS-weighted global efficiency vs age at MRI. (e) Residual of cost-integrated (1-ODI)-weighted global efficiency vs age at MRI. (f) Spearman's partial correlation coefficient of cost-corrected weighted normalised local efficiency and age at MRI. (g) Residual of cost-integrated FS-weighted local efficiency vs age at MRI. ^p<0.01, ^+^p<0.001.

When we compared brain network features at the same level of network density (cost) using a cost-corrected approach and averaged among all levels of network density (cost-integration approach), we observed a significant correlation between binary local efficiency and age at MRI for several levels of network density (>0.35) (Fig. 4A), and after cost-integration (ρ=0.352, p=0.002, Fig. 4B).

When weighting the connections at each level of network density by the average FA and NDI values along the tracts connecting each pair of regions, we observed a positive correlation between age at MRI and the average weighted degree (strength) for FA and NDI-weighted networks (i.e., the average level of FA and NDI in the network at a given density), for all levels of cost assessed (Supplementary Fig. 1), and after cost-integration (ρ=0.658, p<0.001 and ρ=0.699, p<0.001 for FA and NDI respectively, Supplementary Fig. 2). Average 1-ODI weights showed a negative correlation with age at MRI for low network densities (<0.27), and after cost-integration (ρ=−0.293, p=0.012).

Age at MRI was positively correlated with cost-integrated global efficiency (ρ= 0.656, p<0.001 for FA, and ρ=0.713, p<0.001 for NDI weighting) and cost-integrated local efficiency (ρ=0.638, p<0.001 for FA, and ρ=0.681, p<0.001 for NDI weighting) and negatively correlated with cost-integrated (1-ODI)-weighted local efficiency (ρ=−0.360, p=0.002). However, these changes in network efficiency are not necessarily associated with a different network configuration, but may be partially explained by the difference in connectivity weights, which given the formulation of weighted efficiency, alter the shortest path lengths on average.

#### Normalised weighted networks

When using a normalisation approach at each level of network density to assess purely the weighted organisational components of the networks, we observed a high correlation with age at MRI of rFS- and r(1-ODI)-weighted global efficiency for several costs (Fig. 4C) as well as after cost-integration (ρ=0.593, p<0.001 for rFS and ρ=0.390, p<0.001 for r(1-ODI), Fig. 4D-E). Weighted local efficiency, showed a negative correlation with age at MRI for some values of cost (between 0.2 and 0.3) when weighting networks by rFA and r(1-ODI), but for a large range of network densities when weighting by rFS (Fig. 4F). Cost-integrated rFS-weighted local efficiency confirmed this tendency (ρ=−0.584, p<0.001, Fig. 4G).

#### Alternative normalisation: comparison with random equivalent networks

Small-world characteristics obtained by comparison with random equivalent networks showed that despite the negative correlation of local efficiency with age at MRI, rFS-weighted normalised clustering coefficient (γ) was positively correlated with age at MRI (ρ=0.640 p<0.001 Supplementary Material), leading also to an increase in rFS-weighted small-worldness coefficient (ρ=0.272, p=0.020, Supplementary Material).

### Association between network features and degree of prematurity

In this section we show that increasing GA at birth was associated with increased FA and NDI weighted connectivity and increased global and local efficiency. Pure organisational characteristics (normalised weighted networks) showed an association between prematurity and an altered distribution (topology) of relative NDI weights.

#### Raw network features

The analysis of raw network features (obtained from the original reconstructed networks) showed no association between GA at birth and network density, but a positive correlation with median FA and NDI weighted connectivity (ρ=0.250, p=0.033 and ρ=0.231, p=0.049, respectively); FA-weighted global efficiency (ρ=0.251, p=0.032); and local efficiency (ρ=0.247, p=0.035). However, given the strong effect of network density and total connectivity strength on integration and segregation features, it is not clear if these results are also associated with changes to the topological organisation of the network.

#### Cost-corrected networks

**Fig. 5:**
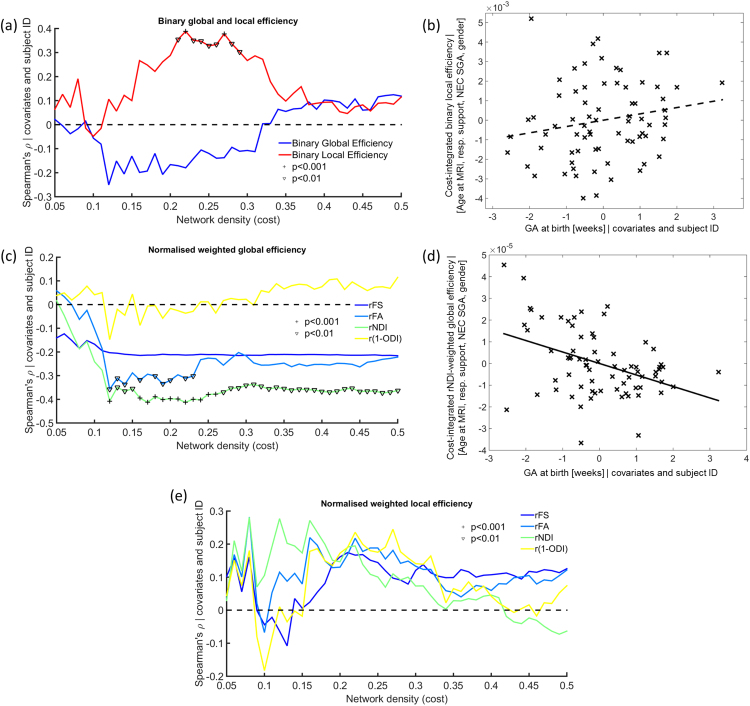
**Association of graph theory features with prematurity (GA at birth).** (a) Spearman's partial correlation coefficient of cost-corrected binary global and local efficiency and GA at birth. (b) Residual of cost-integrated binary local efficiency vs GA at birth. (c) Spearman's partial correlation coefficient of cost-corrected weighted normalised global efficiency and GA at birth. (d) Residual of cost-integrated rNDI-weighted global efficiency vs GA at birth. (e) Spearman's partial correlation coefficient of cost-corrected weighted normalised local efficiency and GA at birth. ^p<0.01, ^+^p<0.001.

Using cost-correction to neutralise differences in network density, the assessment of binary topology of brain networks showed decreased local efficiency associated with increasing prematurity at birth for network densities between 0.21 and 0.29 (Fig. 5A), although the association was non-significant after cost-integration across the whole range of densities (ρ=0.230, p=0.051, Fig. 5B). No significant associations with prematurity were found after directly weighting the cost-corrected networks by FA and NDI values along tracts.

#### Normalised weighted networks

When normalised weighted features were assessed, global efficiency weighted by rNDI showed a significant negative correlation with GA at birth for most network densities (between 0.12 and 0.5, Fig. 5C), as well as after cost-integration (ρ=−0.347, p=0.003, Fig. 5D). Weighting global efficiency by rFA, on the other hand, although also being significantly anti-correlated with GA at birth for a few values of network density, was not significant after cost-integration (ρ=−0.225, p=0.055). We did not observe a significant association between GA at birth and rFA- or rNDI-weighted local efficiency, or with any graph theory characteristic weighted by rFS or r(1-ODI).

#### Alternative normalisation: comparison with random equivalent networks

Using a completely different normalisation approach (comparison with random equivalent networks) we found a similar negative correlation between GA at birth and cost-integrated normalised NDI-weighted global efficiency (ρ=−0.282, p=0.016, Supplementary Material), suggesting that this effect is robust to normalisation approach.

### Core versus non-core connections

The relationship between increased GA at birth and lower rNDI-weighted global efficiency, demonstrates a link between prematurity and network topology weighted by microstructural features. However, from the results in *Association between network features and age at MRI* and *Association between network features and degree of prematurity* the topological alterations underlying this relationship are unclear. In this section we test the hypothesis that this effect is related to the distribution of weights between core and local (non-core) connections.

#### Relationship between core and non-core connections and age at MRI

The median NDI at both core and local connections was directly correlated with age at MRI (Supplementary Fig. 3) for all network densities and after cost-integrating (ρ=0.692, p<0.001 for core connections, and ρ=0.663, p<0.001 for local connections). Note that when normalising NDI weights (rNDI), the sum of all connections is forced to one for each subject, giving more prominence (percentage) to connections with higher NDI values. We observed that median rNDI at core edges was not correlated with age at MRI for most network densities, although it reaches significance after cost-integration (ρ=−0.276, p=0.018). The median rNDI of local edges was positively correlated with age at MRI for certain densities and after cost-integration (ρ=0.328, p=0.005), reflecting changes in the proportion of NDI weighted core/local connections after normalisation.

#### Relationship between core and non-core connections and degree of prematurity

Analogously, when assessing the relationship between core/local NDI-weights with prematurity, we observed that the median NDI in core connections was not significantly correlated with GA at birth, but median NDI-weights of local connections showed a consistent trend for different network densities (Supplementary Fig. 4) and after cost-integration (ρ=0.273, p=0.020), suggesting a variation in core/local NDI weight distribution associated with GA at birth. This difference in core/local NDI distribution (no change in NDI of core connections, but increased NDI in local connections) was clear after normalisation, where we observed that rNDI-weighted core connections were negatively correlated with GA at birth for all network densities as well as when cost-integrated (ρ=−0.382, p<0.001). rNDI-weighted local connections behave in the opposite way, being decreased in preterm babies, i.e., correlated with GA at birth (ρ=0.395, p<0.001). These data suggest that the higher global efficiency observed in normalised NDI networks associated with prematurity might be produced by a decrease in NDI-weights of local connections while maintaining NDI-weights of core key connections that reduce network average shortest path lengths.

### Edge-wise associations in the minimum grid of connectivity

**Fig. 6:**
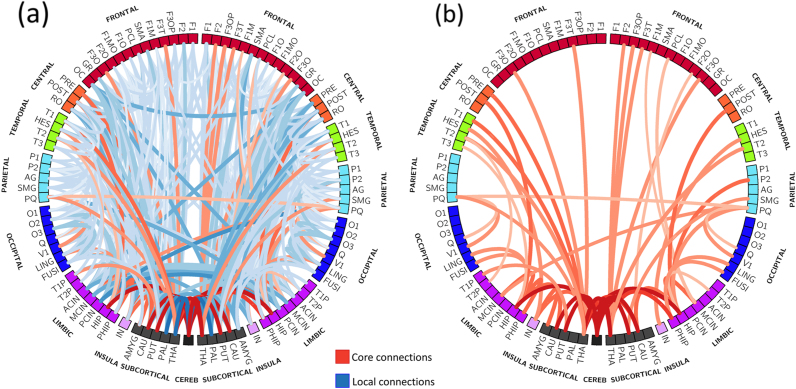
Representation of core and local connections. (a) rNDI-weighted core (red) and local (blue) connections in the minimum grid network (connections common to all subjects). (b) Detail of the rNDI-weighted core connections. Intensity and thickness of the links are proportional to the average rNDI weight. See correspondence of abbreviations with anatomical regions in Supplementary Table 1.

**Fig. 7:**
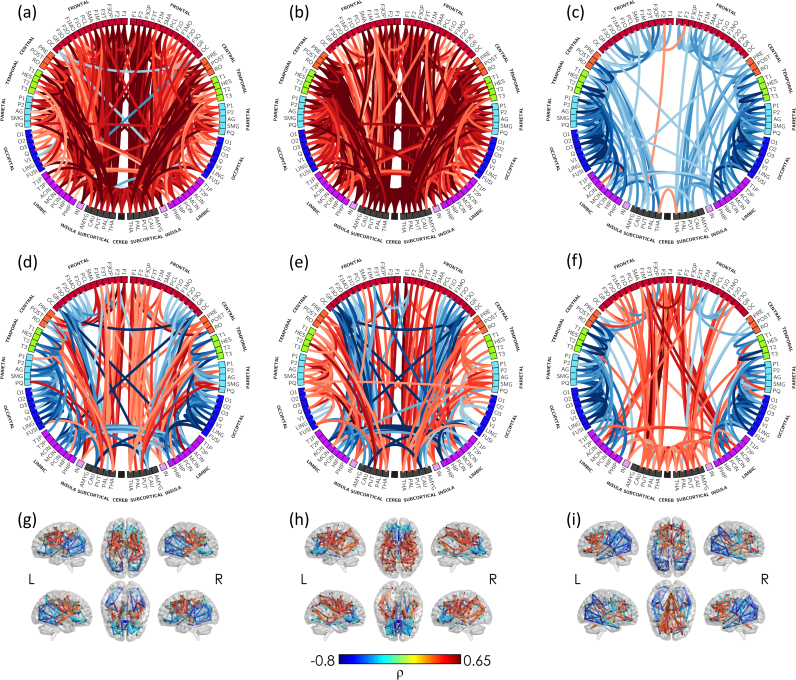
**Edge-wise association with age at MRI in the minimum grid network (connections common to all subjects).** Significant Spearman's partial correlation of (a) FA-, (b) NDI-, (c) (1-ODI)- (d,g) rFA-, (e,h) rNDI- and (f,i) r(1-ODI)-weighted connections with age at MRI (after FDR correction). Intensity and thickness of the links are proportional to the Spearman's partial correlation coefficient (**ρ**). See correspondence of abbreviations with anatomical regions in Supplementary Table 1.

**Fig. 8:**
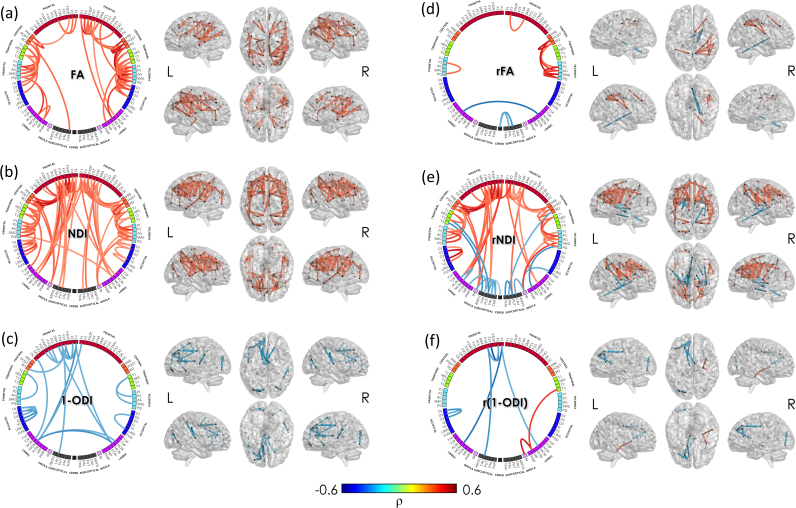
**Edge-wise association with GA at birth in the minimum grid network (connections common to all subjects).** Links show significant Spearman partial correlation of (a) FA-, (b) NDI-, (c) (1-ODI)-, (d) rFA- (e) rNDI- and (f) (1-ODI)-weighted connections with GA at birth (after FDR correction). Intensity and thickness of the links are proportional to the Spearman's partial correlation coefficient (**ρ**). See correspondence of abbreviations with anatomical regions in Supplementary Table 1.

Computation of the connectivity backbone network (“minimum grid” of connections present into all subjects) resulted in a binary network with 505 of the total 4095 connections possible (Fig. 6A). Of note, the rNDI-weighted core of this backbone network included peri-rolandic, some temporal regions, hippocampus, DGM, cerebellum, right precuneus and some short-range occipital connections (Fig. 6B). Assessing the relationship between individual connections and age at MRI highlighted regional variations in maturation (Fig. 7). Assessing partial correlations between network measures and GA at birth enabled those connections most affected by prematurity to be demonstrated (Fig. 8).

#### Association with age at MRI

We observed significant positive correlations between age at MRI (after FDR correction) and WM connectivity weighted by FA and NDI throughout the brain (Fig. 7A-B) and negative correlations between age at MRI and 1-ODI (Fig. 7C). Assessing FA-, NDI-, and (1-ODI)-weighted connectivity in relative terms (rFA, rNDI and r(1-ODI)) enabled regional variations in maturation to be evaluated (Fig. 7D–I). We observed faster increases in relative FA-weighted connectivity (compared with the rest of the connections) in connections between deep grey matter structures and primary motor and somato-sensory cortex, temporal, parietal and frontal cortex. However, relative FA-weighted connectivity in short cortico-cortical connections increased at a slower rate with increasing maturation including those within the frontal lobe, frontal to rolandic operculum, intra-temporal, temporal to parietal, parieto-occipital, intra-occipital, connections between cingulate gyrus and the rest of the brain, and inter-hemispheric connections. Relative NDI-weighted connectivity followed a similar pattern with age at MRI, however, some parietal and occipital connections appeared to mature at a faster rate when assessed by rNDI compared to rFA. Although, 1-ODI generally decreased with age at MRI (in absolute terms), when assessing the correlation of its relative values (r(1-ODI)) with age at MRI, we observed a strong positive association (suggesting a slower rate of decrease compared with the rest of regions) in frontal to deep grey mater structures and contralateral connections, but a negative correlation (faster decrease compared with the rest of regions) between short-range cortico-cortical connections in temporal, parietal, occipital and cingulate regions.

#### Association with degree of prematurity

FA weighted connections that correlated positively with GA at birth included short-range cortico-cortical connections in the frontal and paracentral lobules bilaterally, and in the right parietal lobe; also frontal to postcentral bilaterally, and to right precuneus and cingulate gyrus; postcentral to parietal regions bilaterally, and temporal to parietal and occipital bilaterally (Fig. 8A). NDI-weighted connectivity showed a more wide-spread pattern of reduced connectivity associated with lower gestational age at birth, which included several short range frontal connections bilaterally; frontal cortex to precentral, post-central and rolandic operculum bilaterally; superior temporal gyrus to parietal bilaterally; parietal to post-central gyrus; intra-occipital connections; anterior and middle cingulate gyrus to frontal gyrus ipsi- and contra-laterally; insula to post-central gyrus bilaterally; and several frontal regions to the striatum bilaterally (Fig. 8B). We did not observe any connections that correlated negatively with GA at birth when assessed by absolute FA and NDI weighted connectivity. 1-ODI was only negatively correlated with GA at birth, including several intra-frontal and intra-occipital connections on the left, temporal to supramarginal gyrus bilaterally, contralateral medial and anterior cingulate connections; and cingulate to contralateral frontal cortex (Fig. 8C).

When assessing relative connection strength, a positive correlation with GA at birth in some regions may result in a negative correlation in others, although not all will survive FDR correction. Relative FA weighted connections correlating with GA at birth and surviving FDR correction were sparse. Connections positively correlated with GA had a tendency to be right lateralised, including connections between parietal lobe and post-central and paracentral lobule. Relative NDI-weighted connections positively correlated with GA at birth were mainly in intra-frontal and connections between frontal and peri-rolandic cortex and rolandic operculum on left; frontal to pre- and post-central on right, and frontal to putamen bilaterally; intra-parietal bilaterally and left intra-occipital connections; right rolandic operculum to inferior parietal; medial cingulate to superior frontal bilaterally; anterior cingulate to superior frontal on the left; insula to parietal on the right and to paracentral and supplementary motor area on the left (Fig. 8D). Relative NDI weighted connections that were negatively correlated with GA at birth included those from deep grey matter to temporal cortex (mainly on the left), right pallidum to rolandic operculum, insula to deep grey matter and between DGM structures (Fig. 8E). The association between relative 1-ODI weights and the degree of prematurity was much sparser, only showing a few right sided connections positively correlated with GA at birth, including insula to hippocampus, and hippocampus to temporal lobe. Left intraoccipital and intra-frontal connections, as well as left frontal to caudate and to bilateral medial cingulate were negatively correlated with GA at birth. Importantly, the increase in relative NDI-weights in core connections (see Fig. 6) will result in a reduction of the average shortest path lengths of brain networks in those infants who were born most preterm, resulting in the apparent increase in rNDI-weighted global efficiency demonstrated in the graph theoretical analysis in *Association between network features and degree of prematurity*.

## Discussion

In this study, we assessed the topology of structural brain networks during early development and the impact of prematurity on brain network organisation. We used state-of-the art inference of connectivity using smart filtering of tractography (SIFT), and complemented measures derived from tractography streamline counts and median FA along connections with weighted microstructural features of connectivity between regions. In this way, we not only confirmed some of the topological patterns shown previously to be associated with the rapid changes in the brain occurring during this critical period, but also identified developmental patterns associated with immaturity at birth. We used different normalisation approaches to confirm these results. In particular, by normalising network weights by the total weight of the connectivity matrix at each level of network density for all subjects, we demonstrated differences in graph theory features suggesting alterations in topology, which we showed can be interpreted in terms of alterations in the average weights of core versus local connections. We found that weighting our connectivity networks by microstructural features, in particular NDI, clarified changes in several local connections contributing to an altered global topology of the structural brain network which was related to the degree of prematurity at birth.

### Maturation of WM microstructural features and development of topological organisation

A number of studies have examined brain development from infancy to adolescence using graph theoretical approaches (Baker et al., 2015, Hagmann et al., 2010, Zhong et al., 2016). However, there have been few studies assessing the critical developmental period prior to the time of normal birth (Ball et al., 2014, van den Heuvel et al., 2015). To our knowledge, no studies have used CSD-based tractography or NDI- and ODI-weighted connectivity in order to assess brain network organisation in neonates. The evolution of binary networks with increasing age demonstrated an increase in binary local efficiency, normalised clustering coefficient and small-worldness with age. Although only significant for relatively high network densities (>0.35), which are more prone to include false positives, these results are in line with previous reports (Ball et al., 2014, van den Heuvel et al., 2015). Weighting connectivity by median FA and NDI along tracts showed a clear correlation between average connectivity weights and age at MRI. Importantly, changes in FA were associated with a strong increase in NDI but a decrease in 1-ODI, suggesting that, although the WM is still largely unmyelinated, changes in FA are driven by an increase in neurite density and not by an increase of coherence of the WM fibres. We hypothesise that the decrease in coherence of the fibres (1-ODI) with increasing age is due to the increase of complexity and crossing fibres in the WM.

We observed that both global and local efficiency of raw networks weighted by the fraction of streamlines increased, suggesting that the ability of the brain to exchange information across the network, and its capacity for functional segregation, increases with age at scan. These findings are in line with previous reports of maturation in older children between early childhood and adolescence (Brown et al., 2014, Hagmann et al., 2010, van den Heuvel et al., 2015). When we assessed normalised weighted topology of the fraction of streamlines we observed a positive association between maturation and global efficiency, but a negative correlation with local efficiency (network segregation). These results suggest that, from a topological point of view, long-range connections are increasing at a faster rate than short-range local connections during this period and are consistent with later maturation of cortico-cortical fibres observed on neuronal tracing studies (Burkhalter, 1993).

Graph theory analysis of raw networks showed a general increase in FA- and NDI-weights leading to increased global and local efficiency. However, the pattern of WM microstructure maturation during early development was observed more clearly in terms of individual correlations. Using this approach, we observed that both FA- and NDI-weighted connections were highly correlated with age at MRI in a widespread pattern encompassing most WM connections and 1-ODI weights largely decrease during this period. Assessing *relative* FA, NDI and 1-ODI connectivity allowed us to observe connections maturing at a faster rate (positive correlations) and those maturing more slowly (negative correlations). We observed regional differences in brain maturation, with connections to and from deep grey matter showing most marked developmental changes during this period. Intra-frontal, frontal to cingulum, frontal to caudate and inter-hemispheric connections matured at a slower rate. Of note, the maturational pattern of some intra-hemispheric connections, most notably temporal to parietal and temporal to occipital, showed a slower rate of development when assessed by FA- than by NDI-weighted graphs, and may reflect the impact of crossing fibres on measured FA in these regions, which is supported by our findings of a decrease in 1-ODI weights in these connections. This maturational gradient is consistent with the regional heterogeneity observed histologically with myelination progressing from inferior to superior and from posterior to anterior (Gilles et al., 1983, Yakovlev and LeCours, 1967). Thalamo-cortical and somatosensory tracts mature rapidly during the preterm period and shortly after birth, while myelination of short range cortico-cortical connections is largely post-natal and extends over the first two decades of life (Kostovic and Jovanov-Milosevic, 2006). In addition, dMRI studies in older infants and young children demonstrate that central WM develops earlier than peripheral WM, and occipital WM develops before frontal WM (Dean et al., 2015, Gao et al., 2009).

### Impact of prematurity

We observed a specific pattern of reduced FA and NDI related to the degree of prematurity at birth. Previous reports have demonstrated an association between prematurity and decreased FA values in principal WM paths at term equivalent age (Ball et al., 2010) and in later childhood (Fischi-Gomez et al., 2014, Kim et al., 2014, Pandit et al., 2014). In the present study, we show for the first time that this reduction in FA is associated with a reduction in NDI in the WM throughout the brain. Moreover, after normalisation, we observed a consistent negative correlation of relative NDI-weighted global efficiency with GA at birth, suggesting an alteration in network topology with increased prematurity at birth.

Edge-based correlations identified disrupted FA-, NDI- and (1-ODI)-weighted WM connectivity associated with prematurity in frontal, parietal, precuneus, insula and cingulate regions. NDI weighting identified a more extensive pattern of altered connectivity associated with immaturity at birth including a number of intra-frontal and frontal lobe to cingulum connections. Of note, we did not identify any regions where prematurity was associated with an increase in either FA or NDI weighted or a decrease in 1-ODI connectivity. Studies in older children who were born preterm have demonstrated a similar regional vulnerability in FA weighted connectivity following preterm birth (Fischi-Gomez et al., 2014, Kim et al., 2014, Pandit et al., 2014) and WM connectivity in these regions in childhood is associated with cognitive and behavioural function (Fischi-Gomez et al., 2014).

Although NDI weighted connectivity showed extensive reductions in those most premature infants, by examining the relationship between relative (or percentage) NDI weighting of each connection, we observed that those regions which were most significantly correlated with immaturity at birth were short-range connections in frontal, parietal and occipital regions; frontal and parietal to peri-rolandic and rolandic operculum connections; temporo-parietal connections; connections between frontal lobe and cingulum; and between insula and post-central gyrus. In adult samples these connections have been found to respectively contribute to cognitive efficiency (Gao et al., 2014); specific aspects of language including phonology, semantics, and sentence processing (Vigneau et al., 2006); and social cognition (i.e., the ability to infer the thoughts and beliefs of others) (Mars et al., 2013). Connections between (medial) frontal lobe and cingulate cortex play a significant role in default mode network connectivity (van den Heuvel et al., 2008), which has been implicated in modulating the integration of cognition and emotion (Greicius et al., 2004); and connections between the insula and the primary somatosensory cortex in the integration of emotional responses with bodily homeostasis (Menon and Uddin, 2010, Singer et al., 2009).

Our data suggest that impaired connectivity in regions considered to be important for a wide range of cognitive and behavioural functions are already present in the early neonatal period and may explain, at least in part, impaired neurocognitive and neurobehavioral impairments in this population. These disrupted connections were more evident when weighted by NDI, suggesting this measure may be more sensitive than FA in assessing disruption to WM microstructural development in this population.

### Core versus local connectivity

Assessing the pure topological organisation of the networks highlighted a consistent negative correlation between relative NDI-weighted global efficiency and GA at birth, suggesting an imbalance in core versus local NDI-weighted connectivity. Previous studies in adults, older children and neonates have identified a rich-club of highly interconnected hubs (Ball et al., 2014, van den Heuvel et al., 2015, van den Heuvel and Sporns, 2011), which are considered to be important in facilitating communication between cortical regions. Using a slightly different approach, based on edge centrality in the network, we identified core regions that included some identified in previous rich-club studies. We found that NDI-weights in core connectivity changed little in the period prior to the time of normal birth and were not altered by degree of prematurity. However, increased relative NDI-weighted global efficiency was associated with a reduction of NDI in local connections with increasing prematurity, including connections between thalamus and pallidum to cerebellum; thalamus to superior frontal and supplementary motor regions; interhemispheric connections; wide-spread connections to and from cingulum; and short range intra-lobular connections. These results are in line with our previous work using unweighted network analysis where we demonstrated preserved rich-club connectivity, but diminished peripheral connections in preterm infants compared to term born healthy controls (Ball et al., 2014).

Recent studies in adults have reported a pattern of preserved core-connectivity but disrupted local connectivity in neurological disorders including in those who were born preterm (Fischi-Gomez et al., 2016, Karolis et al., 2016) and in Alzheimer's disease (Daianu et al., 2015). Indeed, in both children and adult survivors of preterm birth, rich-club connectivity is maintained or reinforced, suggesting effective utilisation of a relative paucity of white matter resources by prioritising rich-club organisation over peripheral connectivity (Ball et al., 2014, Fischi-Gomez et al., 2016, Karolis et al., 2016). Our current findings in the neonatal brain of altered NDI weighted edge based and local connectivity associated with prematurity support this hypothesis and suggests that, in the absence of major lesions, the preterm brain is relatively resilient to disruptions to the normal developmental trajectory in core connections and that peripheral connections in regions associated with important aspects of cognition and behaviour including executive function, memory, attention, information processing, response inhibition, salience processing and emotion regulation are more vulnerable following preterm birth.

### Technical considerations

Although the reproducibility of SIFT in neonates has not been assessed, it has been proven to reduce scan–rescan variability and to improve the biological accuracy of the structural connectomes obtained in adults (Smith et al., 2015). Of note, even if we produce relatively dense connectivity matrices, this technique reduces the amount of false positives, which has been shown to be critical for the estimation of network features (Zalesky et al., 2016). In addition, the use of cost-corrected approaches to systematically assess a range of network densities by proportional thresholding the streamlines generated in the original reconstructed networks allowed us to ensure a reduced amount of false positives in lower network densities (which represent the more probable connections, and maximises reproducibility).

## Conclusions

Using brain network characteristics complemented by microstructural characteristics and normalisation approaches, we have shown that brain development prior to term equivalent age is characterised by an increase in FA and NDI, which is particularly rapid in connections between deep grey matter and central cortical regions, and more gradual in frontal regions. This approach was especially useful to highlight the impact of prematurity on network topology when weighted by NDI. Altered network topology in the most preterm infants emerges as a result of an imbalance in core-local connectivity with a reduction of NDI in local connections, especially in short range cortical connections, inter-hemispheric connections and those involving thalamus, cingulum, cerebellum and frontal regions. The relative preservation of core connections may support effective use of impaired white matter reserve following preterm birth.

## Funding

This work was supported by the Medical Research Council (UK) [grant numbers MR/K006355/1 and MR/L011530/1], and the Department of Health through an NIHR Comprehensive Biomedical Research Centre Award (to Guy's and St. Thomas’ National Health Service (NHS) Foundation Trust in partnership with King's College London and King's College Hospital NHS Foundation Trust).

## Figures and Tables

**Table 1:** Clinical characteristics of the infants.

Clinical Characteristic	
Sex (males/females)	41/24
GA at birth (median, range) [weeks+days]	33^+2^ (24^+2^−41^+1^)
PMA at scan (median, range) [weeks+days]	36^+2^ (25^+3^–45^+6^)
More than two days of respiratory support (number, percentage)^a^	22 (34%)
Necrotising Enterocolitis (number, percentage)	7 (11%)
Small for Gestational Age^b^ (number, percentage)	19 (29%)
Twins (number, percentage)	18 (28%)
Extremely preterm (number, percentage)	6 (9%)
Very preterm (number, percentage)	15 (23%)
Preterm (number, percentage)	31 (48%)
Term (number, percentage)	13 (20%)

a Total days requiring mechanical ventilation, continuous positive airways pressure and supplementary oxygen.

b Defined at <10th centile.
