# Supplementary material for: Early development of structural networks and the impact of prematurity on brain connectivity
Source: Neuroimage. 2017 Apr 1;149:379–92. doi: 10.1016/j.neuroimage.2017.01.065 (PMC5387181; doi:10.1016/j.neuroimage.2017.01.065)
Supplement: Supplementary file 1 — Supplementary material [file mmc1.pdf]

Supplementary Material:
Early development of structural networks and the impact of prematurity on brain connectivity

Dafnis Batalle[a], Emer J Hughes[a], Hui Zhang[b], Jaques-Donald Tournier[a], Nora Tusor[a], Paul Aljabar[a], Luqman Wali[a], Daniel C Alexander[b], Joseph V Hajnal[a], Chiara Nosarti[a], A David Edwards[a], and Serena J Counsell[a]

a) Centre for the Developing Brain, Division of Imaging Sciences & Biomedical Engineering, King's College London, SE1 7EH London, United Kingdom b) Computer Science & Centre for Medical Image Computing, University College London, United Kingdom

1. SUPPLEMENTARY RESULTS: SMALL-WORLD NETWORK CHARACTERISTICS

Association with age at MRI:

By means of comparison with binary random equivalent networks maintaining the same size and degree distribution (Maslov and Sneppen, 2002), we obtained small-world coefficients: normalised characteristic path length (α), normalised clustering coefficient (γ) and small-worldness (σ=γ /α) (Humphries and Gurney, 2008; Watts and Strogatz, 1998). α was negatively correlated with age at MRI for a few network densities (around 0.3), while gamma was found positively correlated for higher densities, however, only small-worldness coefficient was significantly associated with age at MRI after cost-integration (ρ=0.268, p=0.022).

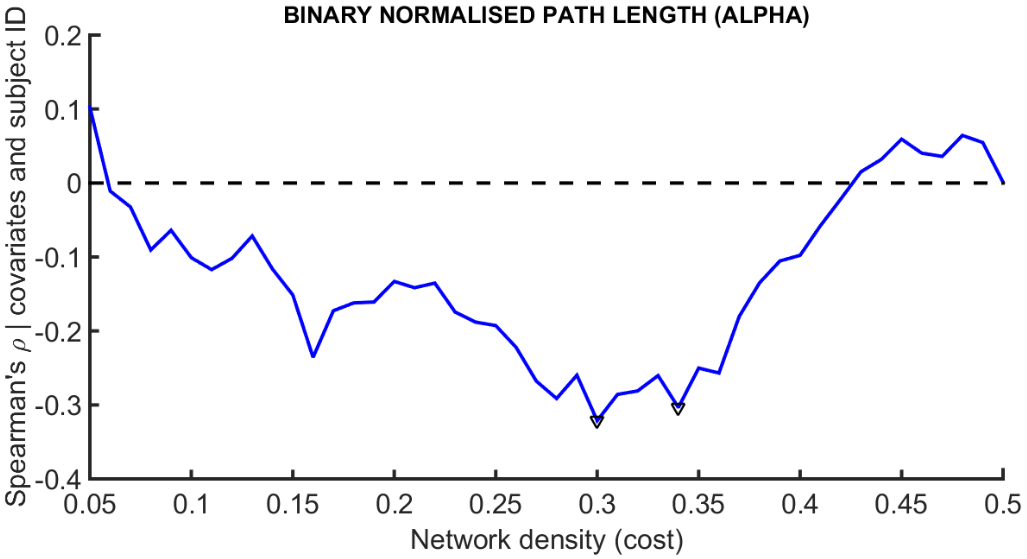

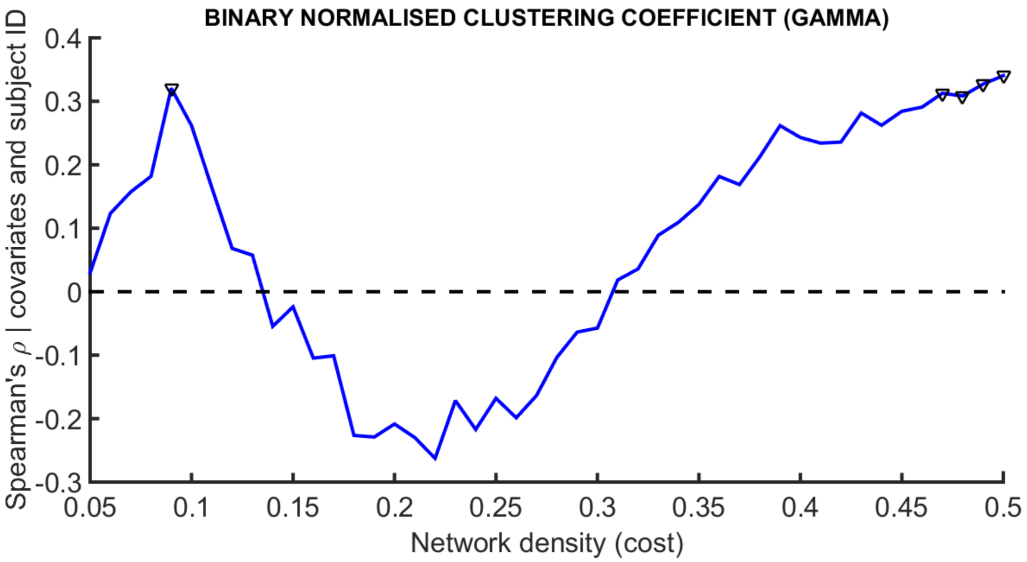
BINARY NORMALISED CLUSTERING COEFFICIENT (GAMMA)
Spearman's ρ | covariates and subject ID
0.4
0.3
0.2
0.1
0
-0.1
-0.2
-0.3
0.05
0.1
0.15
0.2
0.25
0.3
0.35
0.4
0.45
0.5
Network density (cost)

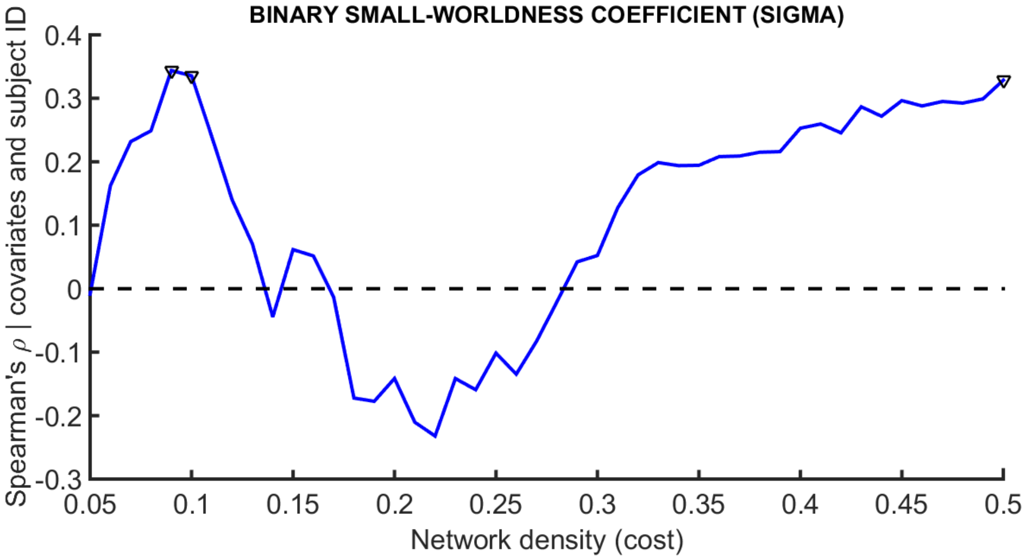
BINARY SMALL-WORLDNESS COEFFICIENT (SIGMA)
Spearman's ρ | covariates and subject ID
0.4
0.3
0.2
0.1
0
-0.1
-0.2
-0.3
0.05
0.1
0.15
0.2
0.25
0.3
0.35
0.4
0.45
0.5
Network density (cost)

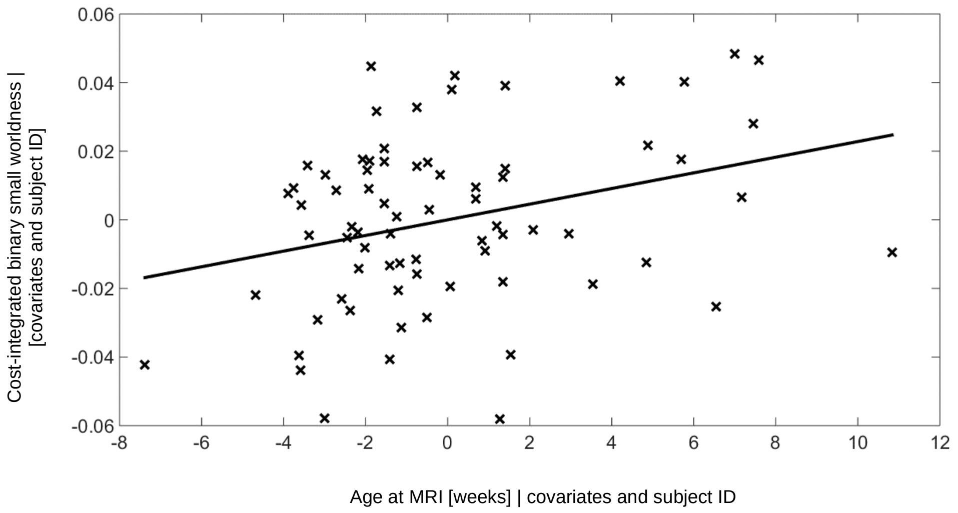

Weighted small-world features also show a significant association with age at MRI. FS-weighted α shows a significant correlation with age at MRI (cost-integrated ρ=0.262 p=0.025), while a negative correlation is found for FA-weighted (cost-integrated ρ=-0.231 p=0.049), NDI-weighted (cost-integrated ρ=-0.261 p=0.026) and (1-ODI)-weighted α (cost-integrated ρ=-0.302 p=0.001). Normalised clustering coefficient (γ) was only found significantly correlated with age at MRI after cost-integration for FS-weighted networks (ρ=0.640, p<0.001). Similarly, small-worldness coefficient (σ) was only significantly correlated for FS-weighted networks (ρ=0.240, p=0.048).

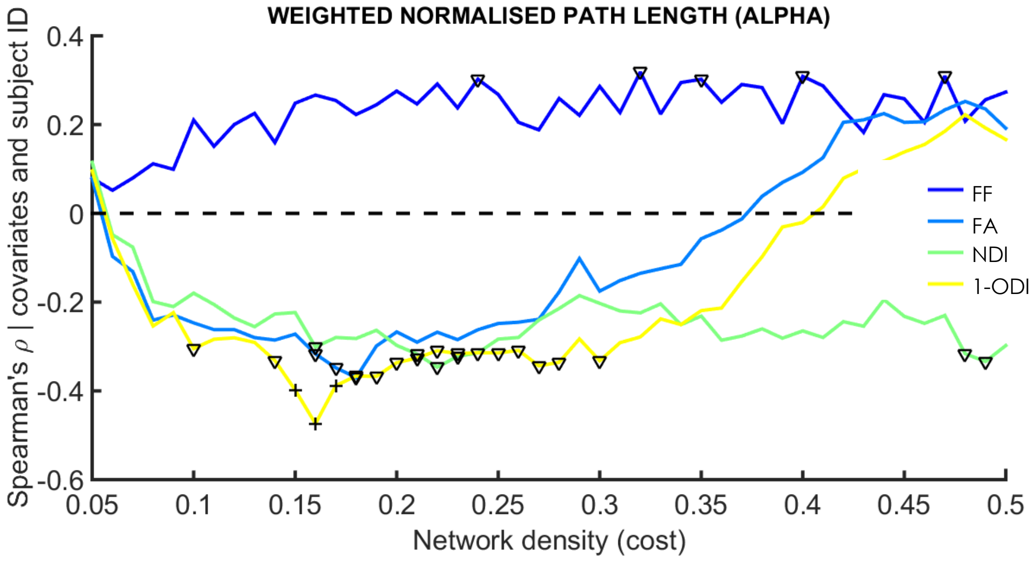

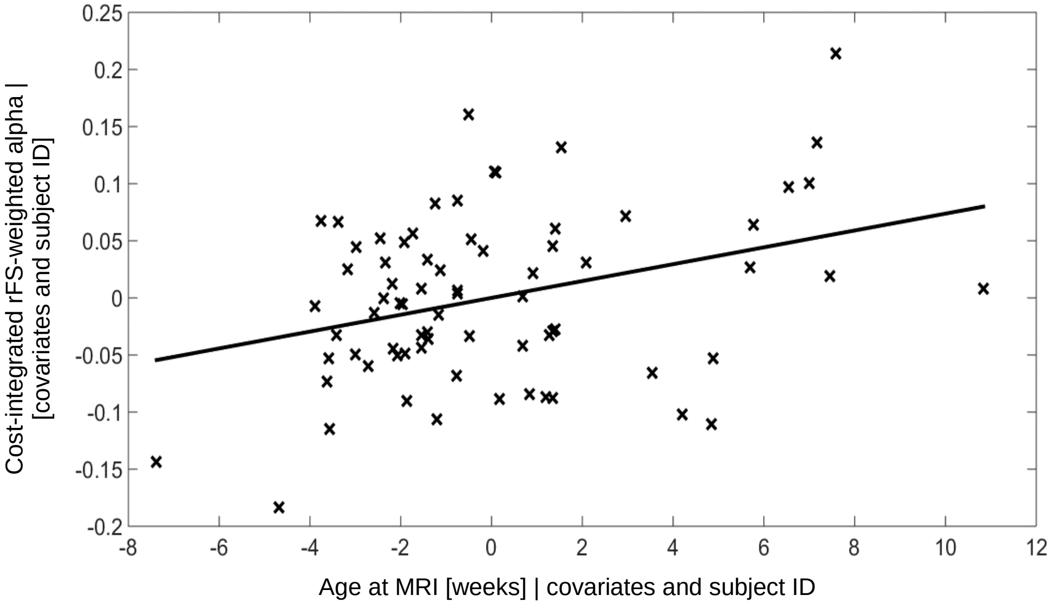

Cost-integrated rFS-weighted alpha | [covariates and subject ID]
Age at MRI [weeks] | covariates and subject ID

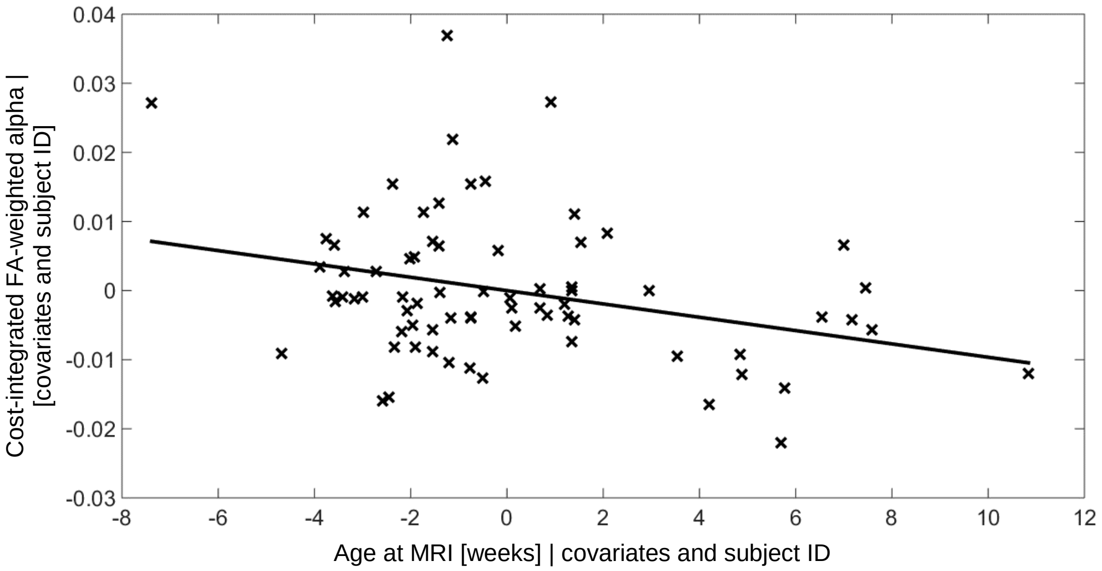

Cost-integrated FA-weighted alpha | [covariates and subject ID]
Age at MRI [weeks] | covariates and subject ID

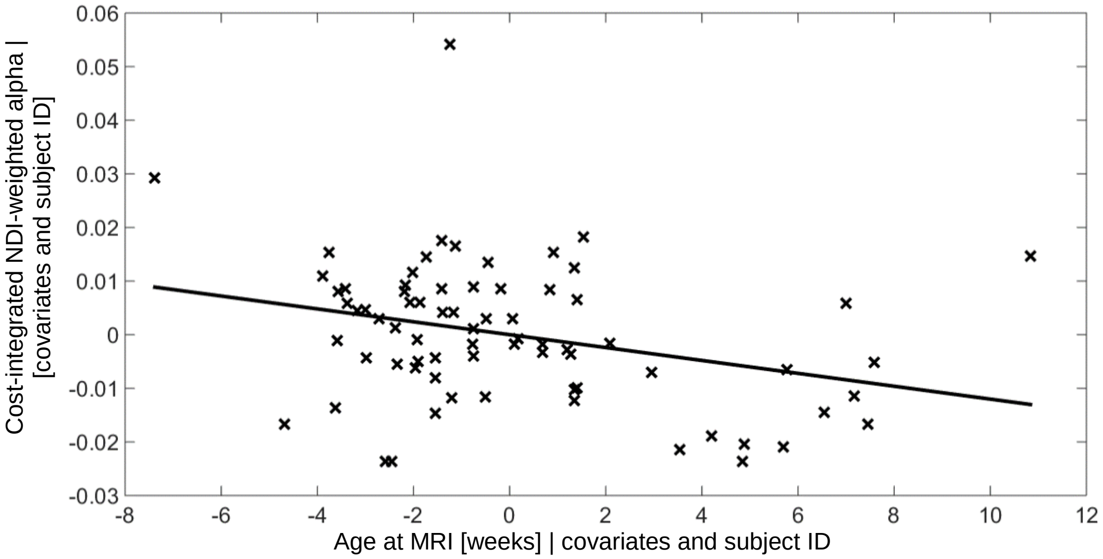
Cost-integrated NDI-weighted alpha | [covariates and subject ID]
Age at MRI [weeks] | covariates and subject ID
0.06
0.05
0.04
0.03
0.02
0.01
0
-0.01
-0.02
-0.03
-8
-6
-4
-2
0
2
4
6
8
10
12

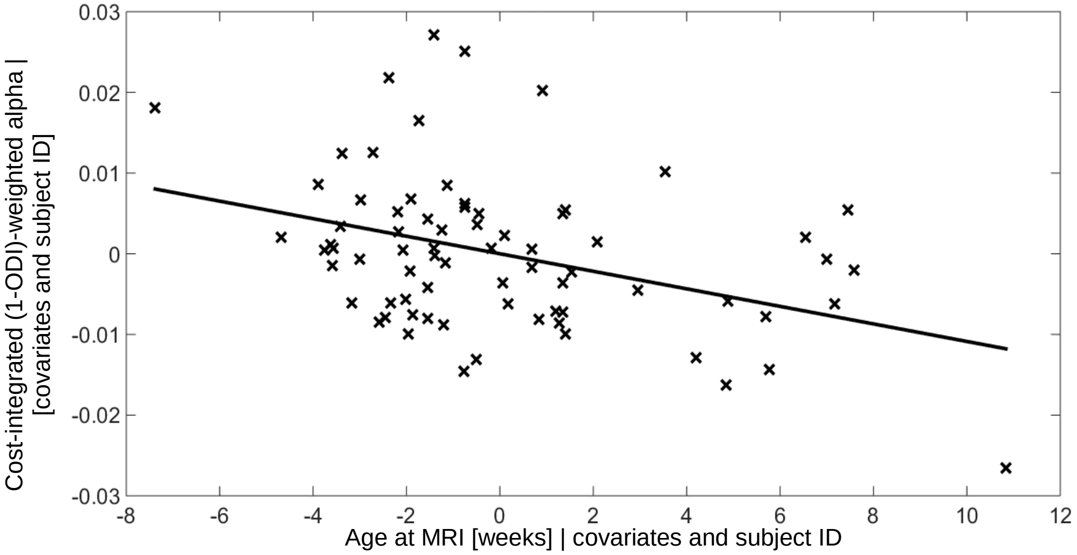
Cost-integrated (1-ODI)-weighted alpha | [covariates and subject ID]
Age at MRI [weeks] | covariates and subject ID
0.03
0.02
0.01
0
-0.01
-0.02
-0.03
-8
-6
-4
-2
0
2
4
6
8
10
12

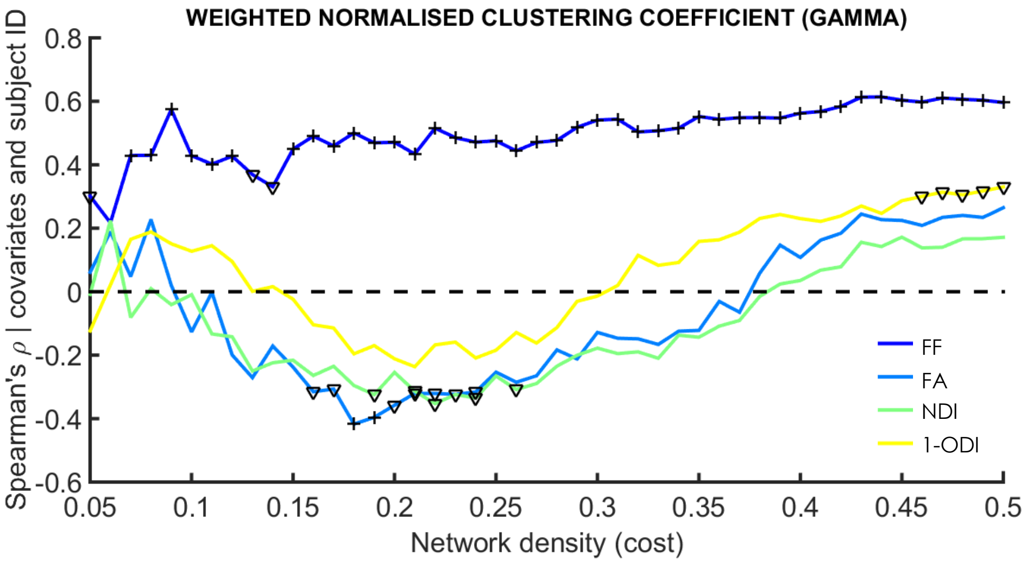
WEIGHTED NORMALISED CLUSTERING COEFFICIENT (GAMMA)
Spearman's ρ | covariates and subject ID
Network density (cost)
FF
FA
NDI
1-ODI
0.8
0.6
0.4
0.2
0
-0.2
-0.4
-0.6
0.05
0.1
0.15
0.2
0.25
0.3
0.35
0.4
0.45
0.5

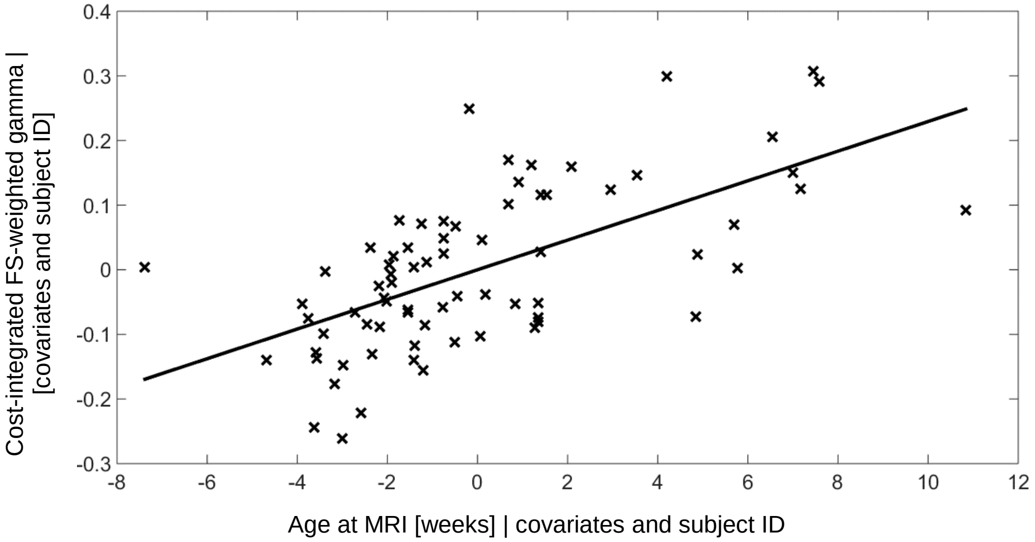
Cost-integrated FS-weighted gamma | [covariates and subject ID]
Age at MRI [weeks] | covariates and subject ID
0.4
0.3
0.2
0.1
0
-0.1
-0.2
-0.3
-8
-6
-4
-2
0
2
4
6
8
10
12

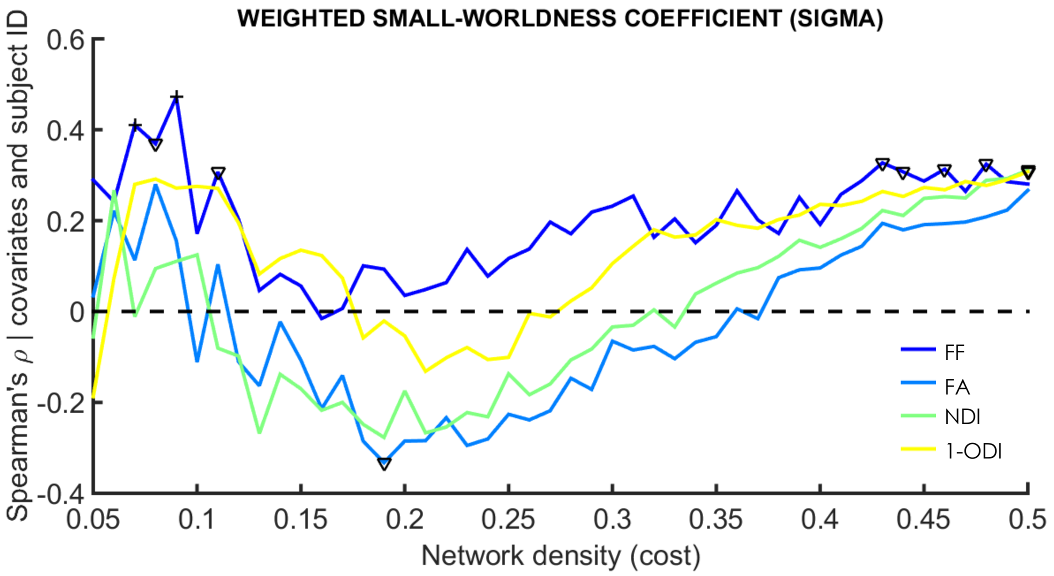
WEIGHTED SMALL-WORLDNESS COEFFICIENT (SIGMA)
Spearman's ρ | covariates and subject ID
Network density (cost)
FF
FA
NDI
1-ODI

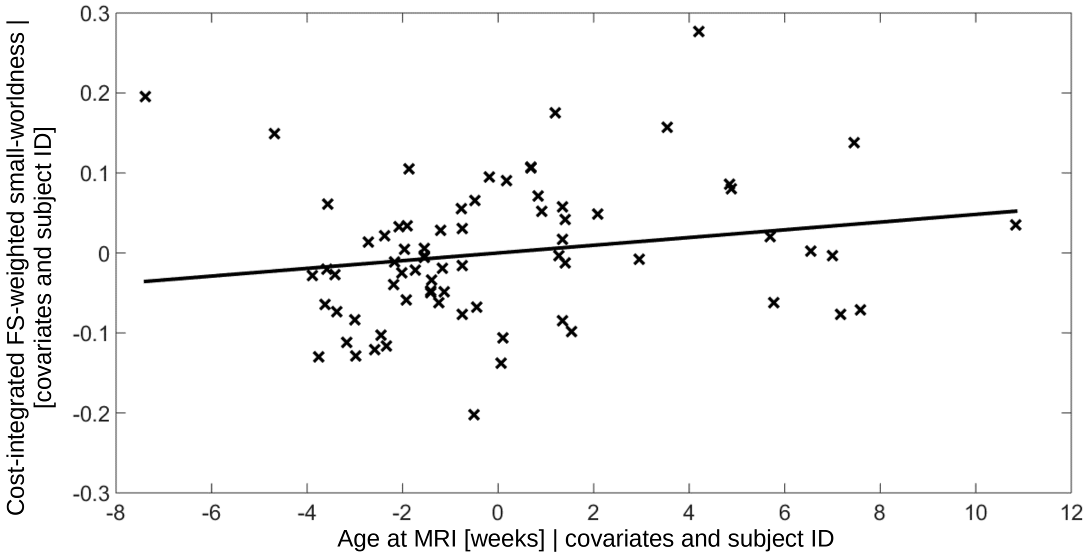
Cost-integrated FS-weighted small-worldness | [covariates and subject ID]
Age at MRI [weeks] | covariates and subject ID

Association with prematurity (GA at birth):

Partial correlation of binary small-world features with age at birth was non-significant, only showing some tendency towards significance with cost-integrated γ (ρ=0.202, p=0.087).

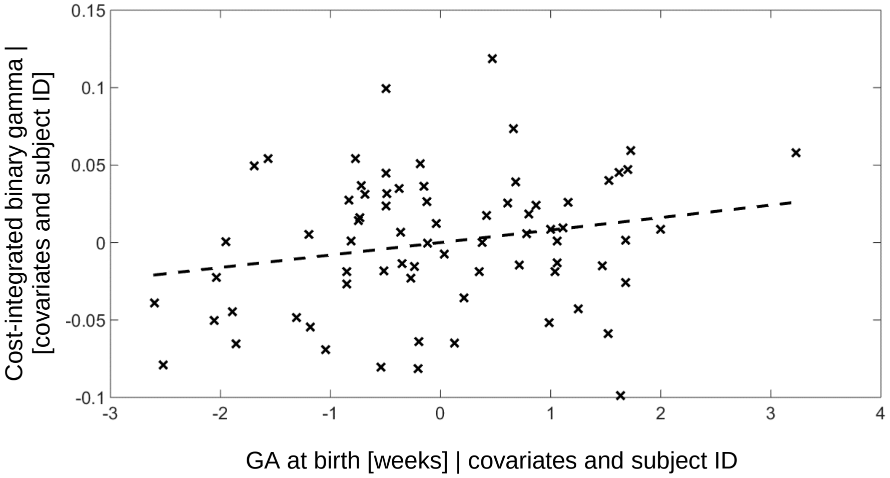

Association of weighted small-world features with prematurity was significant for NDI-weighted α at several network densities (between 0.1 and 0.25) and after cost-integration (ρ=0.285, p=0.015). Consistently, weighted global efficiency normalised (divided) by the global efficiency of random equivalents was negatively correlated with age at birth for a similar range of network densities and after cost-integration (ρ=-0.342, p=0.004).

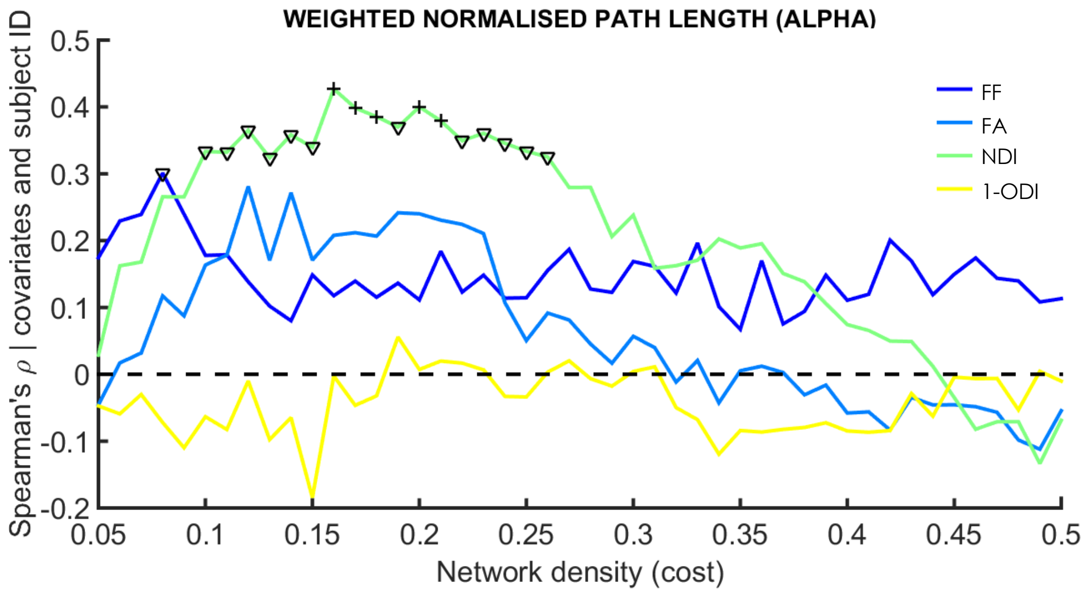
WEIGHTED NORMALISED PATH LENGTH (ALPHA)
Spearman's ρ | covariates and subject ID
Network density (cost)
FF
FA
NDI
1-ODI
0.5
0.4
0.3
0.2
0.1
0
-0.1
-0.2
0.05
0.1
0.15
0.2
0.25
0.3
0.35
0.4
0.45
0.5

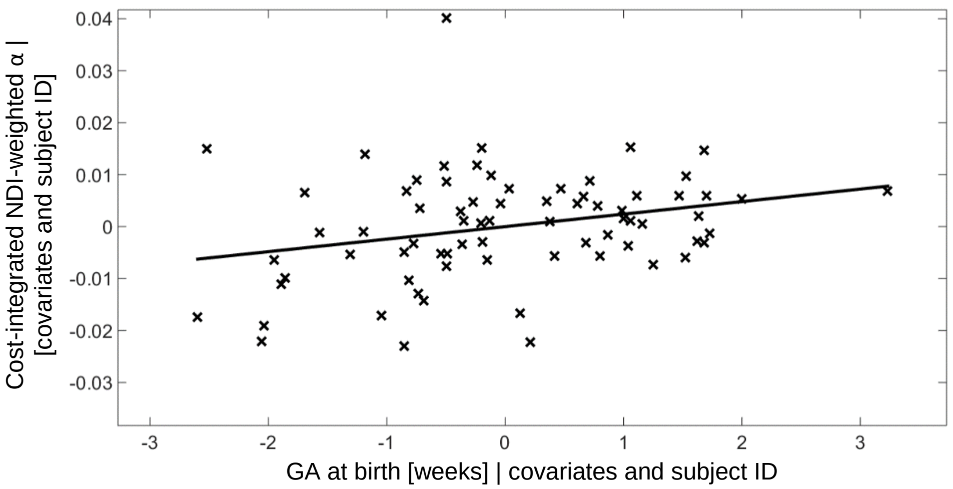
Cost-integrated NDI-weighted α | [covariates and subject ID]
GA at birth [weeks] | covariates and subject ID
0.04
0.03
0.02
0.01
0
-0.01
-0.02
-0.03
-3
-2
-1
0
1
2
3

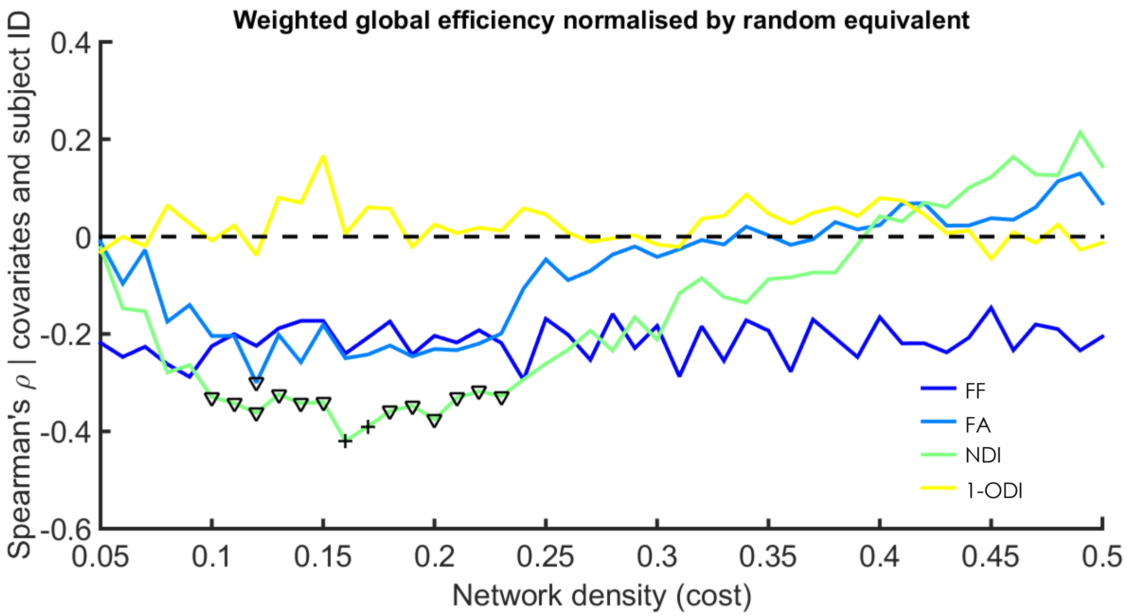
Weighted global efficiency normalised by random equivalent
Spearman's ρ | covariates and subject ID
Network density (cost)
0.4
0.2
0
-0.2
-0.4
-0.6
0.05
0.1
0.15
0.2
0.25
0.3
0.35
0.4
0.45
0.5
FF
FA
NDI
1-ODI

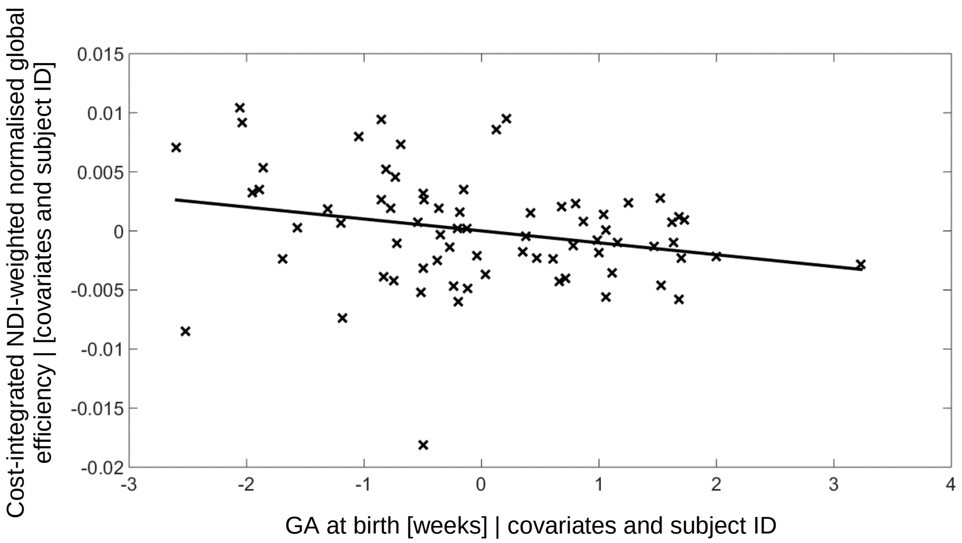
Cost-integrated NDI-weighted normalised global efficiency | [covariates and subject ID]
GA at birth [weeks] | covariates and subject ID
0.015
0.01
0.005
0
-0.005
-0.01
-0.015
-0.02
-3
-2
-1
0
1
2
3
4

NDI-weighted normalised clustering coefficient (γ), was weekly associated with age at birth, but remained significant after cost-integration (ρ=0.321, p=0.006). No significant association was found with small-worldness coefficient.

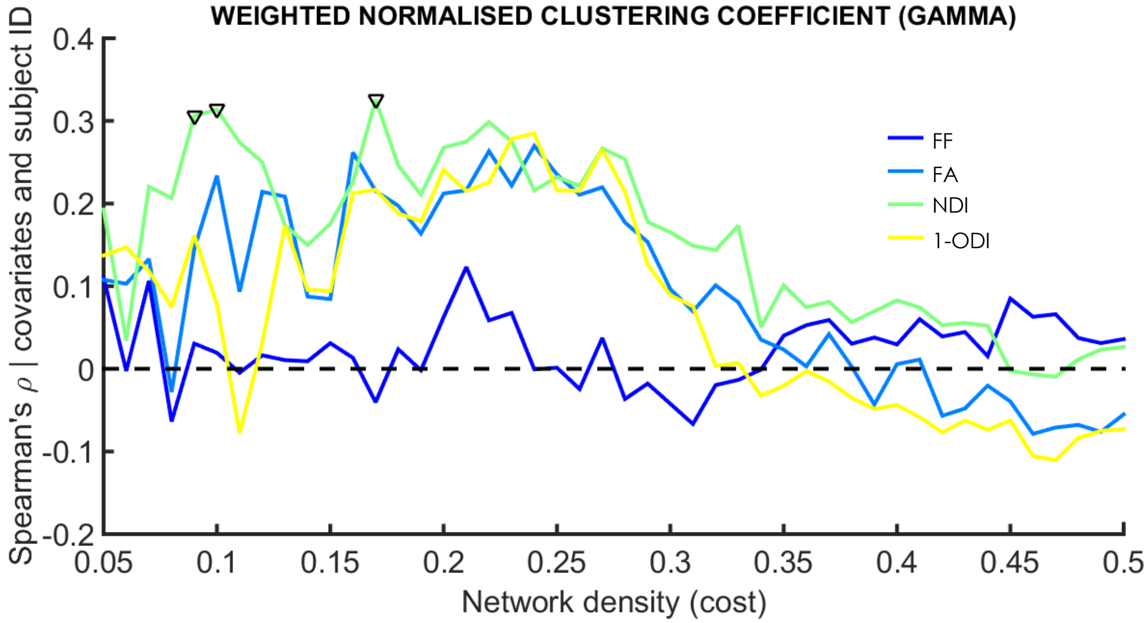

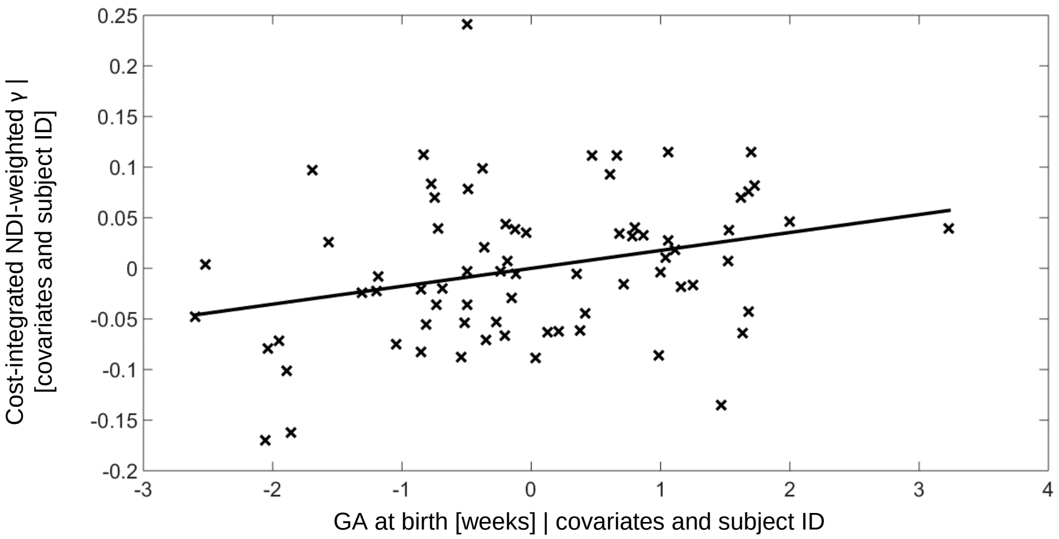

REFERENCES

Humphries MD, Gurney K. 2008. Network 'small-world-ness': a quantitative method for determining canonical network equivalence. PLoS One 3:e0002051.
Maslov S, Sneppen K. 2002. Protein interaction networks beyond artifacts. FEBS Lett 530:255-256.
Watts DJ, Strogatz SH. 1998. Collective dynamics of 'small-world' networks. Nature 393:440-442.

2. SUPPLEMENTARY FIGURES

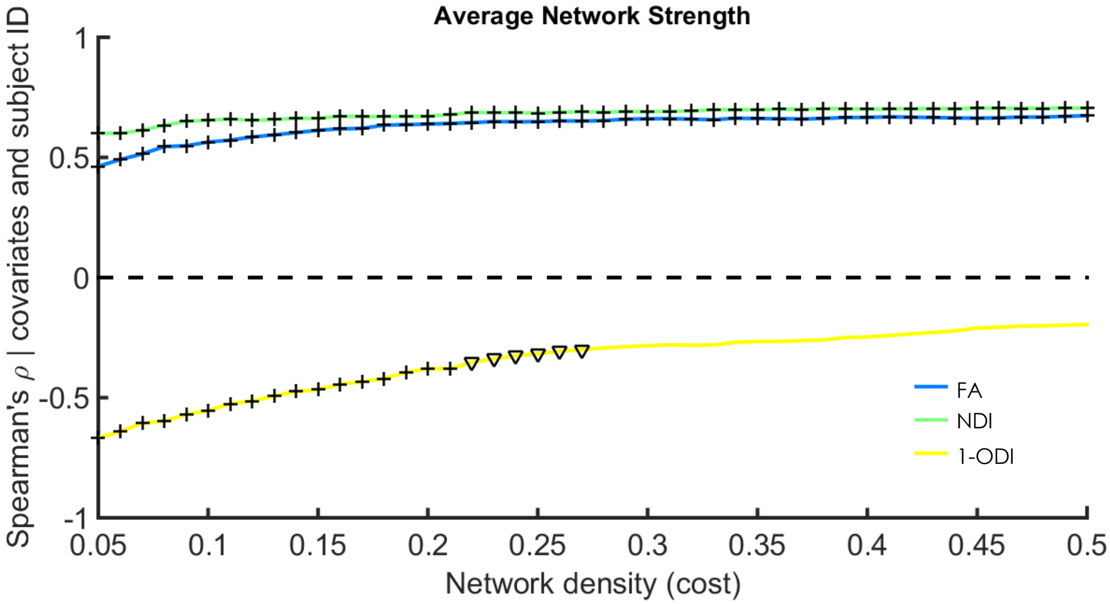

Supplementary Figure 1. Spearman's partial correlation of average FA- and NDI-weighted degree and age at MRI at different levels of network density.

+p<0.001, ^p<0.01

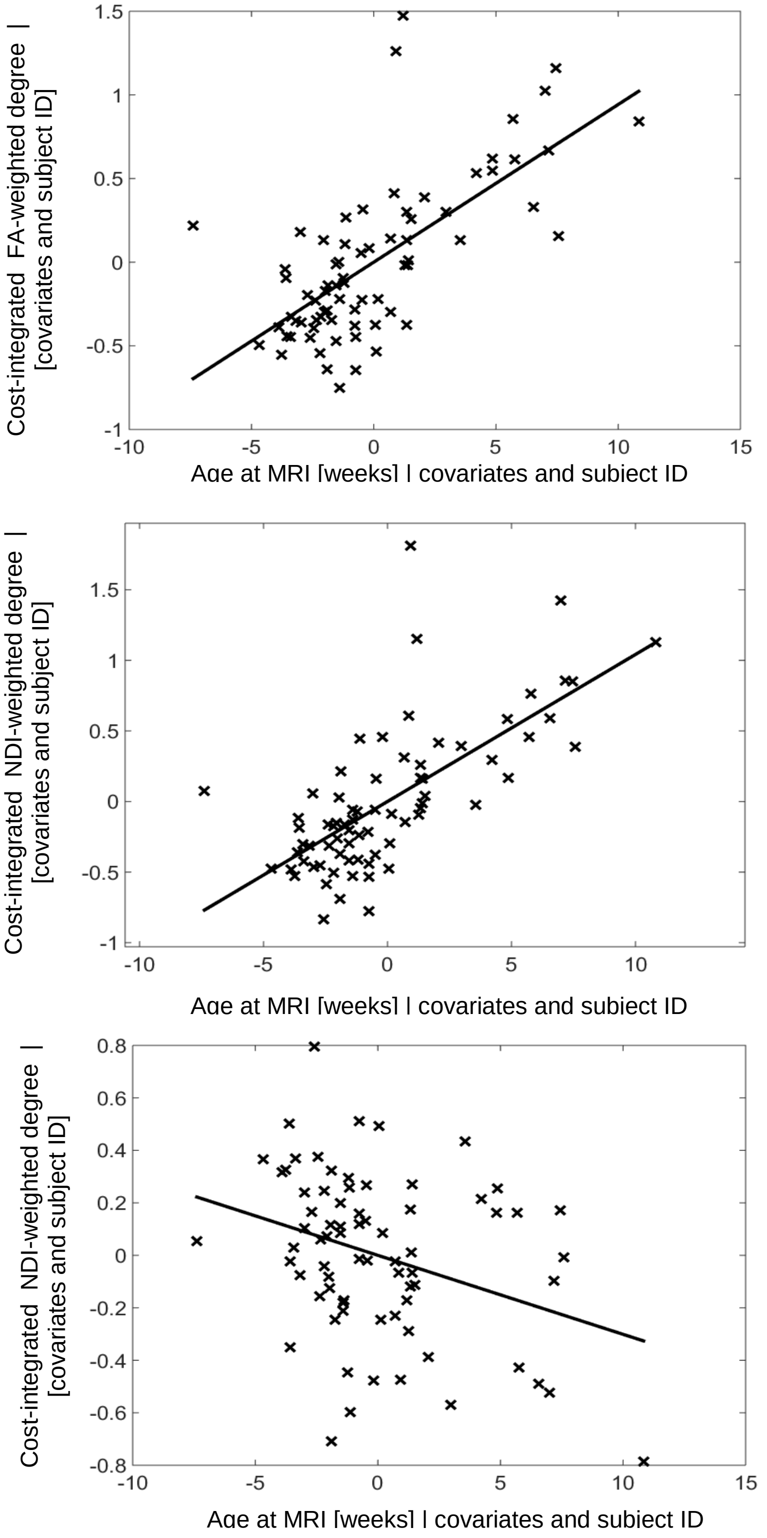

Supplementary Figure 2. Spearman's partial correlation of cost-integrated average FA- and NDI-weighted network degree and age at MRI

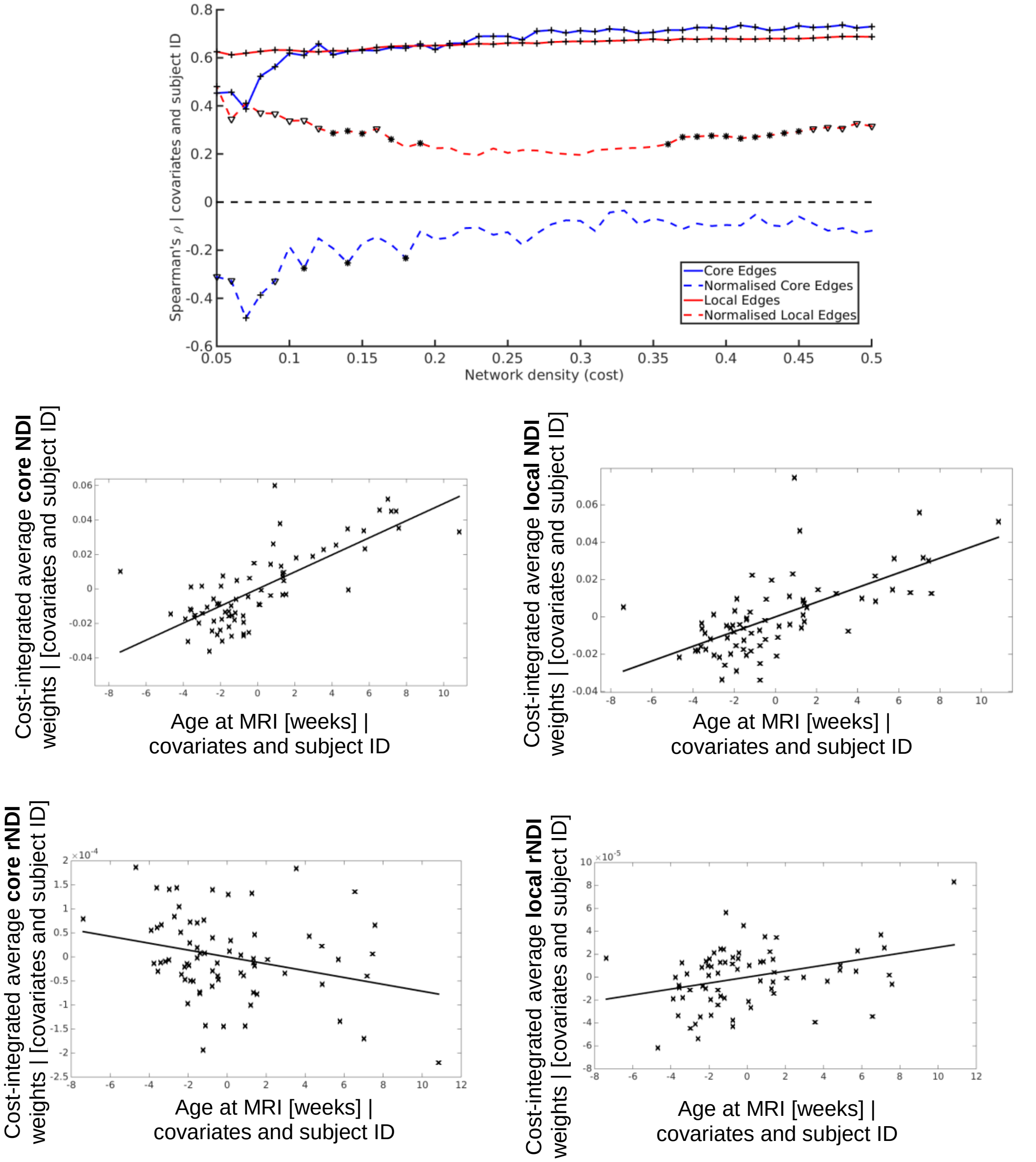

Supplementary Figure 3. Spearman's partial correlation of median NDI and rNDI weights of core and local connections with **age at MRI**, and significant cost-integrated results+p<0.001, ^p<0.01, *p<0.05

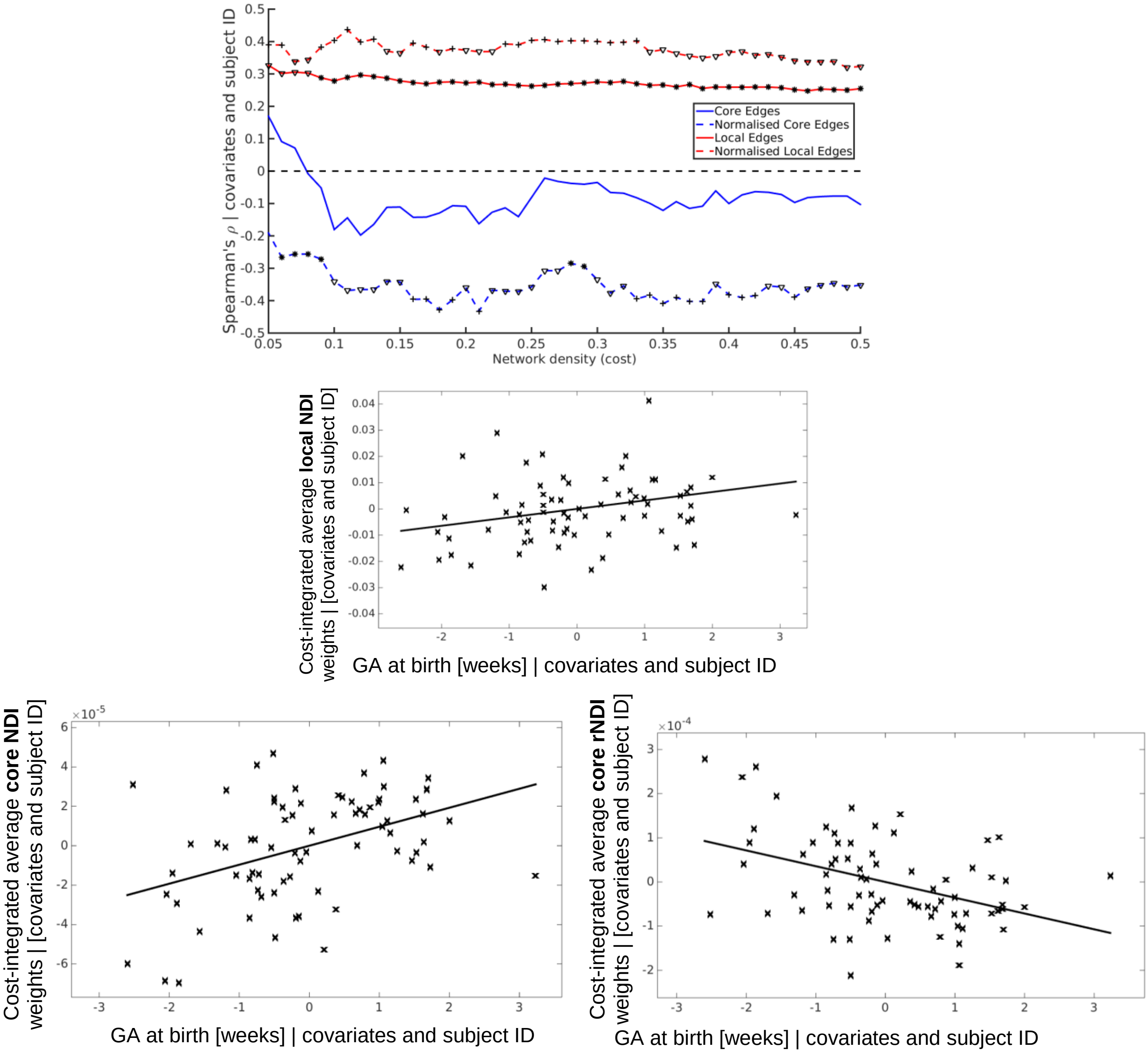

Supplementary Figure 4. Spearman's partial correlation of median NDI and rNDI weights of core and local connections with **GA at birth**, and significant cost-integrated results

+p<0.001, ^p<0.01, *p<0.05
